# Proteomics-Driven Mechanistic Insights into the Anti-Inflammatory Potential of Thinned Apple Polyphenols in a DNBS-Induced Colitis Model in Mice

**DOI:** 10.1021/acs.jproteome.5c00653

**Published:** 2026-01-26

**Authors:** Giulio Ferrario, Daniela Impellizzeri, Giovanna Baron, Ramona D’Amico, Giulio Fumagalli, Tommaso Gnasso, Ezio Bombardelli, Marina Carini, Rosanna di Paola, Giancarlo Aldini, Alessandra Altomare

**Affiliations:** † Department of Pharmaceutical Sciences (DISFARM), 9304 Università degli Studi di Milano, Via Mangiagalli 25, 20133 Milano, Italy; ‡ Department of Chemical, Biological, Pharmaceutical and Environmental Sciences, 220297 University of Messina, Viale F. Stagno D’Alcontres 31, 98166 Messina, Italy; § Plantex S.a.s., Galleria Unione 5, 20122 Milano, Italy; ∥ Department of Veterinary Sciences, University of Messina, Viale SS Annunziata, 98168 Messina, Italy

**Keywords:** Nrf2, anti-inflammatory, antioxidant, polyphenols, proteomics, thinned apples

## Abstract

Ulcerative colitis (UC) is a multifactorial inflammatory bowel disease (IBD) with increasing incidence worldwide. Current treatments, including NSAIDs and corticosteroids, provide partial symptom relief but are associated with significant side effects, highlighting the need for novel therapies with improved safety profiles. Given the role of oxidative stress and inflammation in driving tissue damage during colitis, natural compounds with antioxidant and anti-inflammatory properties represent promising therapeutic candidates. Thinned apples (TA), an agricultural byproduct, were identified as a valuable source of polyphenols (TAP) with demonstrated anti-inflammatory and antioxidant activities in a cell-based inflammation model. This study evaluates TAP’s therapeutic potential in a DNBS-induced colitis mouse model using label-free quantitative proteomics. Proteomic analysis revealed modulation of key pathways affected by TAP treatment, including: (i) activation of antioxidant defense mechanisms; (ii) reversal of DNBS-induced alterations, specifically ferroptosis and heme-toxicity; (iii) suppression of immune responses; and (iv) attenuation of ulcerative features, with downregulation of proteins involved in coagulation, inflammation, and angiogenesis. Overall, TAP showed significant therapeutic effects by targeting oxidative stress and inflammation, supporting its use as a polyphenol-rich extract in health products for UC. Moreover, repurposing TA as a bioactive extract offers an innovative strategy for industrial applications in therapeutic development.

## Graphical abstract

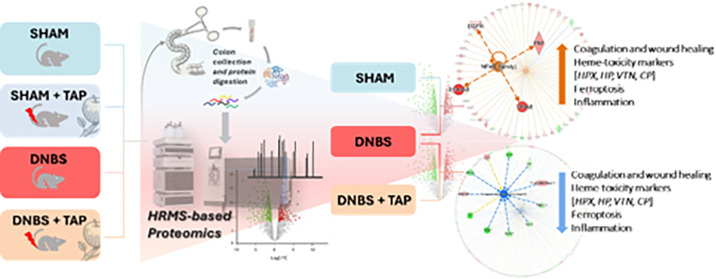

## 1 Introduction

Inflammatory bowel disease (IBD) is characterized by chronic inflammation of the gastrointestinal (GI) tract, with ulcerative colitis (UC) being one of the main forms, specifically affecting the colon. The incidence of UC has significantly increased in recent decades, greatly impacting patients’ quality of life due to alternating periods of remission and exacerbation^1^ Although its etiology is unclear, a common hypothesis involves defects in tight junctions leading to increased intestinal permeability and antigen entry, contributing to inflammation. Current treatments, including aminosalicylates and corticosteroids, are symptomatic and associated with significant side effects.^2^ Biologics directed against key inflammatory mediators (e.g., anti-TNF-α monoclonal antibodies) have demonstrated substantial efficacy in inducing and maintaining remission in IBD, but their use is constrained by rigorous eligibility criteria in clinical trials and by high treatment costs which restrict patient access, especially in resource-limited settings.^3–5^ Thus, the need for new therapeutic strategies with reduced side effects remains a priority. Given the role of oxidative stress and inflammation in UC tissue damage, natural compounds with antioxidant and anti-inflammatory properties are being explored as potential treatments.^6–8^

Plant-derived natural extracts have long been valued as sources of bioactive molecules, and the pharmaceutical industry has recently revisited them, particularly for polyphenols shown to benefit gut health in animal models and humans. Phytoconstituents can target two key cellular systems: the Nrf2/ARE and NF-κB pathways^9,10^ Nrf2/ARE, a central defense mechanism against oxidative stress, controls antioxidant gene expression, while NF-κB is the main effector pathway in inflammation, promoting pro-inflammatory genes and exacerbating mucosal inflammation in IBD.^11^ Apples are a rich source of phytochemicals, particularly polyphenols, which can induce Nrf2 due to their chemical structure. Certain 1,2-diphenols present in apples and their derivative products (e.g., quercetin and its glycosides, procyanidins and their monomers catechin and epicatechin, and chlorogenic acid) can promote Nrf2 activation by interacting with cysteine residues of the Keap1 regulatory protein, when oxidized to their more reactive quinone form. Emerging evidence also suggests that apple phytochemicals inhibit NF-κB, reducing inflammation.^12^ This dual action suggests potential use as dietary supplements to improve gut health. In this regard, thinned apples, while representing a huge waste in the apple production chain as they make up 65–70% of the ripening fruit, are an interesting source of polyphenols, given their content more than 10 times higher than in ripe apples. In a recent work conducted by our research group, the polyphenol profile of thinned apples was obtained and their anti-inflammatory and antioxidant potential was demonstrated on cellular models.^12^

The crucial results gathered from the *in vitro* studies laid a solid background for subsequent *ex-vivo* studies aimed at evaluating the therapeutic potential of orally administered thinned apple polyphenolic extract (TAP) in an animal model of UC (DNBS-induced UC). Besides macroscopic and microscopic assessment of the extent of tissue damage (on explanted colon) upon TAP treatment, using state-of-the-art techniques in the field of proteomics, functional studies were performed to describe at the molecular level the distinctive biological processes of the pathological phenotype, as well as to understand the molecular pathways evoked by TAP extract to effectively limit the inflammatory state. Even though there is general knowledge of the potential targets of these compounds, omics approaches could better illustrate the biological impact of the extract and identify the most relevant phytochemical compounds. Combining the use of these advanced analytical strategies with a circular economy concept to valorize industrial waste products could reduce the impact of cultivation on waste production, minimizing disposal problems while obtaining a potential source of bioactive compounds. This work specifically could help in promoting the use of thinned apples as a valuable source of apple polyphenols to be used in health care products to prevent/treat oxidative and inflammatory chronic conditions.

## 2 Material and Methods

### 2.1 Reagents

Purified and sterilized bidistilled water, obtained using the Milli-Q system (Millipore, Bedford, MA), was used for buffer preparation and other required applications. For the animal studies, rodent chow was obtained from Envigo (Teklad, Milan, Italy), isoflurane from Piramal Critical Care (Voorschoten, Netherlands), DNBS from Sigma-Aldrich (Milan, Italy), and hematoxylin and eosin (H&E), ethanol, and paraffin were sourced from Bioptica (Milan, Italy). The thinned apple polyphenol extract (TAP), investigated for its anti-inflammatory activity and potential commercialization, was provided by Plantex Research Srl (Milan, Italy). The reagents for tissue homogenization included SDS (sodium dodecyl sulfate) from Bio-Rad (Hercules, CA), NaCl (sodium chloride) and MgCl2 (magnesium chloride) from Fluka Chemical (Ronkonkoma, New York), TEAB (triethylammonium bromide), Benzonase Nuclease, and cOmplete protease inhibitor cocktail, all sourced from Sigma-Aldrich (Milan, Italy) and Roche Diagnostics GmbH (Mannheim, Germany), respectively. For the proteolytic digestion of protein extracts, solutions containing TCEP (tris­(2-carboxyethyl) phosphine), IAA (iodoacetamide), and AMBIC (ammonium bicarbonate) were also prepared using reagents from Sigma-Aldrich. Additional reagents used during digestion included phosphoric acid (H_3_PO_4_) from Fluka Chemical, methanol (MeOH), and TEAB (previously mentioned). Trypsin (sequencing-grade) was supplied by Roche. For LC-MS analysis, ultrapure formic acid (FA) and acetonitrile (ACN) were sourced from Sigma-Aldrich.

### 2.2 Animal Model

#### 2.2.1 General Characteristics

Male CD1 mice (25 g; Envigo, Milan, Italy) were housed in a well-ordered locality (room 22 ± 1 °C 12-h dark/light cycles) with ordinary rodent chow and water. Messina University Review Board for the animal care endorsed the research. All animal experiments were conducted in accordance with institutional guidelines and were approved by the Animal Welfare Body (OPBA) of University of Messina and by the Italian Ministry of Health, in compliance with Italian legislation (D.Lgs. 26/2014) and EU Directive 2010/63/EU (Ethics Approval No. 956/2024-PR).

#### 2.2.2 Induction of Colitis, Treatment with TAP and Colon Sampling

Colitis was provoked in mice by an intrarectally injection of DNBS as previously indicated.^13–15^ Briefly, mice were anesthetized with isoflurane; DNBS (4 mg in 100 μL of 50% ethanol per mouse) was instilled into the rectum inside a catheter introduced 4.5 cm proximal to the anus. Thereafter, the animals were kept for 15 min in a Trendelenburg position to avoid reflux. On day 4 after DNBS injection, the animals were weighed and sacrificed, the abdomen opened by a midline cut. The colon was removed and freed from surrounding tissues, opened along the antimesenteric border, washed, weighed and processed for histological and biochemical studies.

#### 2.2.3 Experimental Design

Animals were randomly divided into several groups (*n* = 6 for each group) (Figure 1):

1. Sham + vehicle group (**SHAM**): Vehicle alone (100 μL of 50% ethanol) was administered intrarectally in control (sham) instead of DNBS and treated orally with saline by oral gavage for 4 days.
2. Sham + TAP extract (10 mg/kg dissolved in saline) (**SHAM_TAP**): Vehicle alone (100 μL of 50% ethanol) was administered intrarectally in control (sham) instead of DNBS and treated orally with TAP by oral gavage for 4 days.
3. DNBS + vehicle group (**DNBS**): Mice were subjected to DNBS administration described as above, and saline was administered by oral gavage every 24 h, for 4 days, starting from 1 h after the administration of DNBS.
4. DNBS + TAP extract (10 mg/kg) group (**DNBS_TAP**): Mice were subjected to DNBS administration described as above, and TAP (10 mg/kg) was administered by oral gavage every 24 h, for 4 days starting from 1 h after the administration of DNBS.

**Figure 1:**
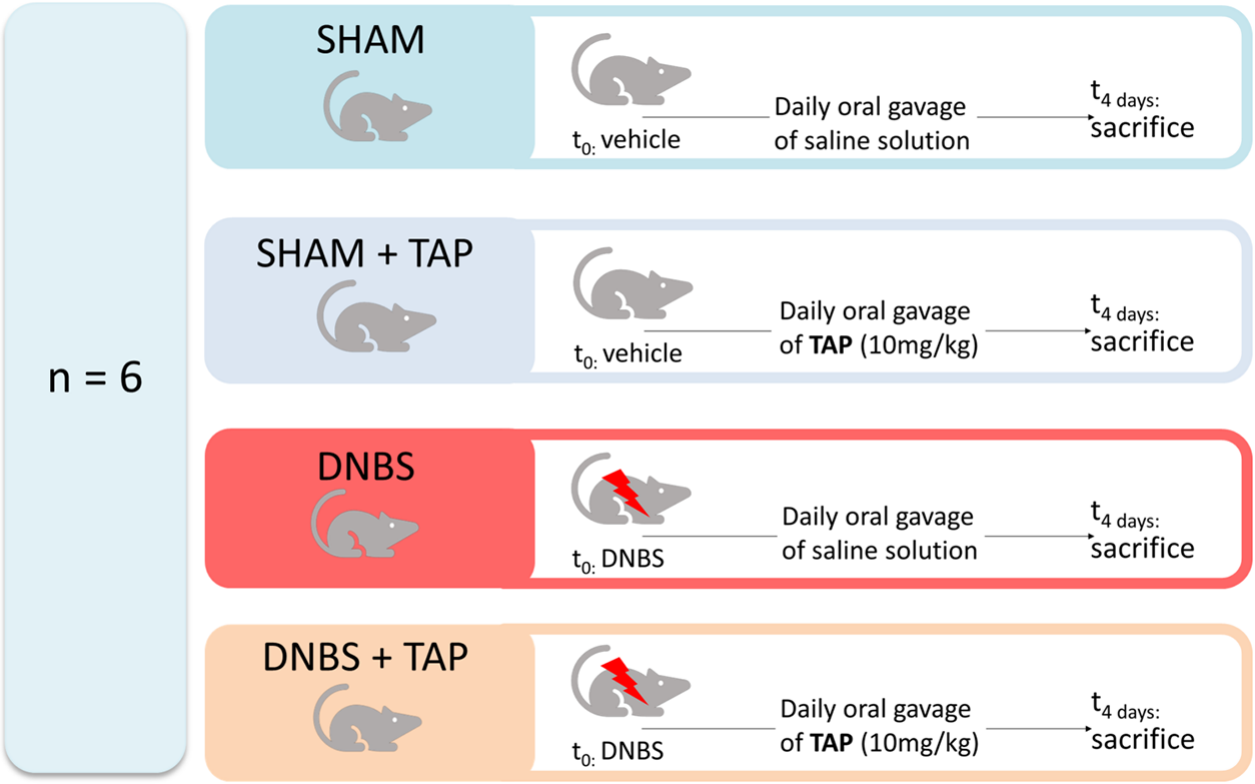
Animal treatment scheme.

Since no significant change was found between sham groups, we presented data of sham + vehicle groups only. The dose of TAP extract was chosen based on previous study.

#### 2.2.4 Evaluation of Colon Damage

After its removal, the total colon was softly washed with saline, opened by a longitudinal incision, and examined under a microscope. Macroscopic damage score was evaluated and scored by two independent observers. 0, no injury; 1, limited hyperemia without ulcers; 2, linear ulcers and no important inflammation; 3, linear ulcers with one site of inflammation; 4, two or more sites of inflammation with ulceration covering >1 cm along the length of the colon; and 5–8, additional one point for each centimeter of ulceration beyond an initial 2 cm.^13–15^

#### 2.2.5 Histological Examination

For histological analysis, tissues were collected, fixed in 10% of buffered formalin phosphate, embedded in paraffin sectioned and subjected to hematoxylin and eosin staining. The degree of histological damage of the colon sections was scored semiquantitatively from 0 to 4 as described. In particular: 0, no indication of inflammation; 1, very little level of inflammation; 2, little level of leucocytes infiltration; 3, elevated level of leucocytes infiltration, elevated vascular density, colon wall thickening; 4, loss of goblet cells, transmural infiltration elevated vascular density and colon wall thickening. Five H&E stained sections from each mouse were scored in a blinded fashion, using a Leica DM6 microscope (Leica Microsystems SpA, Milan, Italy) associated with Leica LAS X Navigator software (Leica Microsystems SpA, Milan, Italy).^13–15^

#### 2.2.6 Myeloperoxidase Assay

Neutrophil infiltration in the colon was monitored by measuring tissue myeloperoxidase (MPO) activity using a spectrophotometric assay with tetramethylbenzidine as substrate as previously indicated.^15^

### 2.3 Label-Free Quantitative Proteomics (LFQ)

#### 2.3.1 Sample Preparation - Tissue Homogenization and Proteolytic Digestion

The tissue homogenization process used a specific harsh lysis buffer to dissolve hard-to-solubilize proteins, compatible with S-Trap devices used later for proteolysis (SDS 5%, TEAB 50 mM, NaCl 50 mM, MgCl2 5 mM, Benzonase Nuclease 500 U/mL, cOmplete cocktail). Tissue samples were homogenized following the procedure described by Manoni, Altomare et al.^16^ Protein concentration was assessed for each sample using the BCA kit (provided by Sigma-Aldrich) according to the manufacturer instructions, and 30 μg of protein extract were processed through tryptic digestion using a recent technology, the S-Trap spin-columns (Protifi, Huntington, New York) as reported by Ferrario et al.^17,18^ In brief, proteins solubilized in SDS-containing buffer were reduced with TCEP, alkylated with iodoacetamide, acidified, and precipitated before loading onto S-Trap columns in methanol-containing buffer. Following extensive washing to remove contaminants, on-column digestion with sequencing-grade trypsin was performed under optimized conditions. Resulting peptides were efficiently recovered through sequential elution with acetonitrile/water/formic acid buffers, dried under vacuum, and stored at – 80 °C until LC–MS analysis.

#### 2.3.2 nLC-HRMS Analysis: Orbitrap Fusion Tribrid Mass Spectrometer

The nLC-HRMS (*nano Liquiq Chromatography-High Resolution Mass Spectrometry*) method, optimized and routinely applied in our laboratories, was performed using a Dionex Ultimate 3000 nano-LC system (Sunnyvale, CA) coupled to an Orbitrap Fusion Tribrid high-resolution mass spectrometer (Thermo Scientific, Bremen, Germany). Each sample was analyzed in triplicate (three technical replicates) as previously reported by Ferrario et al.^17,18^ with 100% acetonitrile washes performed between runs to prevent carry-over. For each injection, 5 μL of peptides were loaded onto an Acclaim PepMap C18 column (75 μm × 25 cm, 100 Å pores) protected by a precolumn (100 μm × 2 cm, 100 Å pores), thermostated at 40 °C. Peptide separation was achieved using a 117 min linear gradient (1–40% acetonitrile/0.1% FA), followed by rinsing and re-equilibration, for a total run time of 144 min. MS analysis was performed with a nanospray ionization source in positive ion mode, operating in data-dependent acquisition (DDA). Full MS spectra were acquired in the Orbitrap at 120,000 resolution, while the top 10 precursors were fragmented in the LTQ by collision-induced dissociation (CID). Dynamic exclusion, charge state screening, and monoisotopic precursor selection were enabled.

#### 2.3.3 Data Analysis

The acquired data were analyzed using MaxQuant software (v.1.6.0; Max Planck Institute of Biochemistry, Germany), with protein identification performed by means of Andromeda search engine.^18^ MaxQuant employs a target–decoy strategy for false discovery rate (FDR) estimation, maintaining an FDR below 1% at the PSM, peptide, and protein group levels. PSM identifications were filtered at 1% FDR based on posterior error probability from the concatenated target–decoy search, and peptide identifications were filtered accordingly. At the protein level, a 1% FDR threshold was applied following MaxQuant’s default inference rules, including subset grouping, assignment of razor peptides, and the Occam’s razor principle. Protein identification was carried out against the UniProt *Mus musculus* reference proteome (Taxonomy ID: 10090). Trypsin was specified as the digestion enzyme, allowing up to two missed cleavages and a maximum of five modifications per peptide. Methionine oxidation and protein N-terminal acetylation were set as variable modifications, while cysteine carbamidomethylation was defined as a fixed modification. Proteins identified with at least two peptides were considered for quantification. Label-free quantification (LFQ) was performed using the MaxLFQ algorithm, with the match-between-runs option enabled and other parameters kept at default settings. Statistical analysis was subsequently performed with Perseus software (v.1.6.1.43; Max Planck Institute of Biochemistry, Germany). LFQ intensities were log_2_-transformed, and values obtained from technical replicates were averaged within each biological replicate prior to statistical analysis, ensuring that differential expression testing was performed exclusively on biological replicates and avoiding artificial inflation of sample size. Differential protein expression was assessed by two-sample *t* tests with Benjamini–Hochberg correction at an FDR of 0.05. Proteins with an adjusted *p*-value (*q* < 0.05) and supported by at least two peptides were considered significantly regulated. The reproducibility of biological replicates was evaluated by calculating Pearson correlation coefficients from LFQ intensities.^18^

#### 2.3.4 Pathway and Network Analysis

Pathway enrichment and protein–protein interaction analyses were performed using STRING (version v.1.7.0) and Ingenuity Pathway Analysis (IPA, QIAGEN, September 2021). STRING analysis was conducted using default parameters, integrating Reactome, KEGG, and Gene Ontology Biological Process repositories. Protein–protein interaction networks were generated based on high-confidence associations derived from experimental data, coexpression, and curated databases. Enrichment significance was assessed using the Benjamini–Hochberg false discovery rate (FDR < 0.05).

IPA was employed to identify canonical pathways, molecular interaction networks, and potential upstream regulators significantly associated with the differentially expressed proteins. The activation or inhibition states of canonical pathways and regulators were predicted using the activation z-score algorithm, which evaluates the consistency between observed protein expression changes and known causal relationships reported in the literature. The directionality of molecular interactions within each pathway was used to infer functional activation patterns, with positive z-scores indicating activation and negative z-scores indicating inhibition.

In both STRING and IPA analyses, the enrichment background was restricted to the set of proteins experimentally identified in our data set, thereby ensuring that enrichment statistics reflected the actual experimental detection space rather than the entire theoretical proteome. All analyses were performed using the *Mus musculus* reference database.

### 2.4 Targeted ELISA Validation of Proteomic Findings

For targeted validation of the proteomic results, clusterin and haptoglobin were selected as biomarker candidates for ELISA-based quantification. These two proteins were chosen based on their differential expression observed in the proteomics data set and their potential functional relevance to the ulcerative colitis disease phenotype, as well as to the mechanism of action of the TAP extract treatment. Colon tissue samples for ELISA analysis were obtained from the murine model of DNBS-induced ulcerative colitis (with and without treatment with thinned apple polyphenol extract) and processed as described in the proteomic sample preparation section, using a lysis buffer containing SDS to homogenize the tissues. In order to remove residual SDS and ensure accurate ELISA measurements, the tissue homogenates were purified using Pierce Detergent Removal Spin Columns (Thermo Scientific, Waltham, MA) according to the manufacturer’s protocol. The detergent-free lysates were then reassessed for protein concentration using BCA Protein Assay Kit (Sigma-Aldrich, St. Louis, MO) to normalize sample loading. Clusterin and haptoglobin levels in the cleared homogenates were quantified by sandwich ELISA. A mouse Clusterin ELISA Kit and a mouse Haptoglobin ELISA Kit were used for the respective targets (both kits from Aviva Systems Biology, San Diego, CA). Both ELISAs were performed strictly according to the kit instructions, and the analyte concentrations in each sample were determined by interpolation from the standard curves generated with the provided kit standards.

## 3 Results and Discussion

### 3.1 Effect of TAP Extract Administration on Macroscopic and Microscopic Alterations, Neutrophil Infiltration and Animal Body Weight Increase in DNBS Injected Mice

No macroscopic alterations were observed in the colons of the sham group. In contrast, 4 days after the intrarectal administration of DNBS, the colons appeared fragile and exhibited signs of mucosal congestion, watery stool, and ulcerations (Figure 2A). Oral administration of TAP at a dose of 10 mg/kg significantly reduced inflammation in DNBS-treated mice compared to the sham group (Figure 2A). Furthermore, all DNBS-treated mice experienced weight loss compared to the sham group, although oral TAP mitigated this loss (Figure 2B). Overall, TAP treatment alleviated the clinical signs of colonic inflammation (Figure 2A,B). Histological analysis showed no structural alterations in the colon tissue of sham mice (Figure 2D and corresponding histological score, panel G). In contrast, DNBS-treated mice exhibited pronounced necrosis, edema, and leukocyte infiltration (Figure 2E and corresponding histological score, panel G). Oral TAP administration significantly reduced these histological alterations (Figure 2F and panel G). Neutrophil infiltration, assessed through the MPO assay, was markedly increased in DNBS-treated mice compared to sham controls (Figure 2H). However, TAP treatment effectively decreased MPO activity, indicating reduced neutrophil infiltration (Figure 2H).

**Figure 2:**
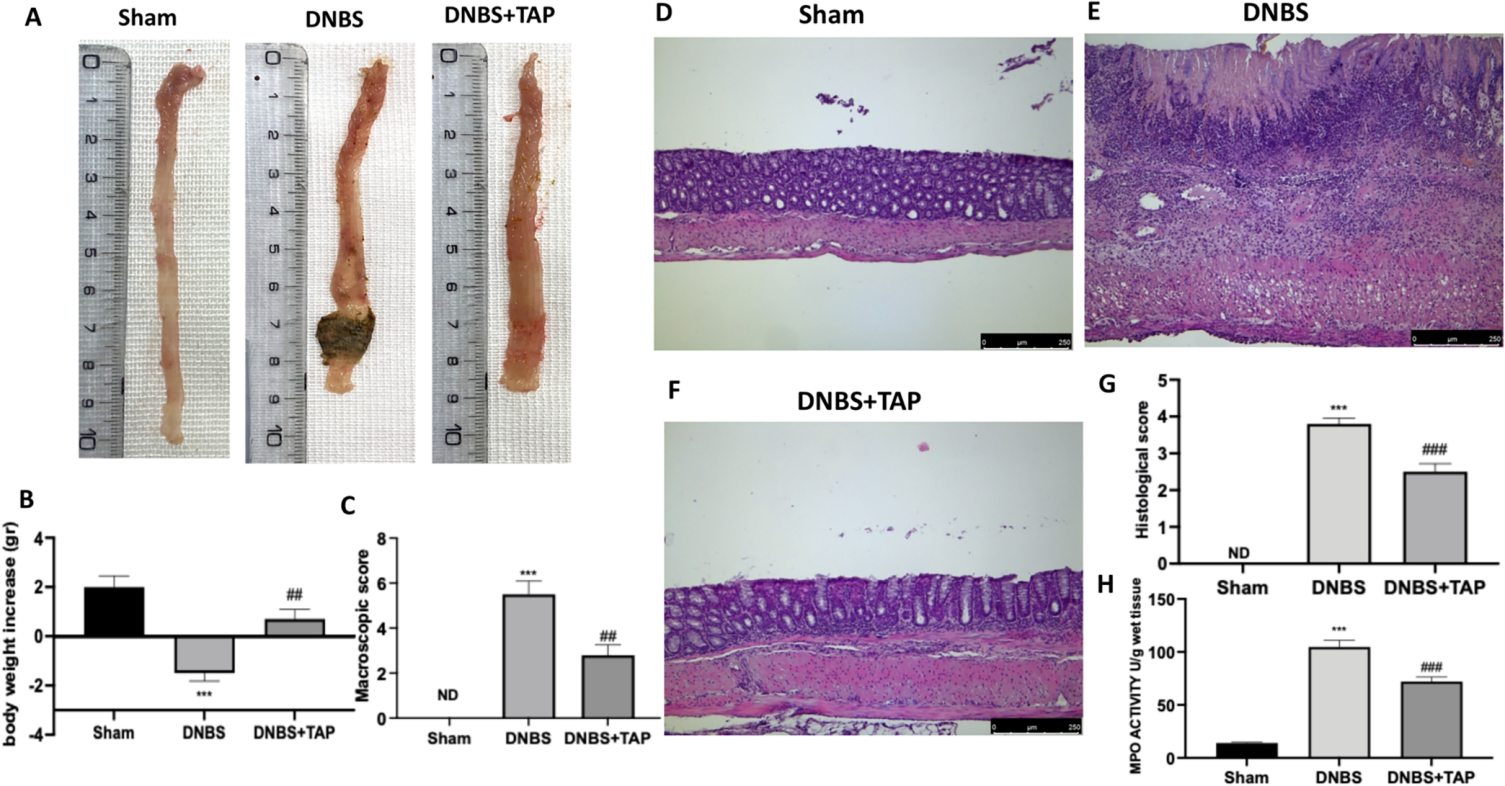
Representative macroscopic picture of SHAM, DNBS and DNBS+TAP groups (A), body weight increase in gr (B) and macroscopic score graph (C). Representative microscopic photos of histological sections of colons in sham (D), DNBS (E) and DNBS+TAP groups (F) with histological score (G). MPO assay measurement (H). Figures are representative of three independent stainings performed on different days. Magnification 10×, scale bar 250 μm. Data are means ± SEM of 6 mice for each group. ***P < 0.001 vs SHAM; ^##^ *P* < 0.01 vs DNBS. ^###^ *P* < 0.001 vs DNBS. ND: not detectable.

### 3.2 Quantitative Proteomics (LFQ)

#### 3.2.1 Data Elaboration and Overall Analysis

Three comparison matrices were built to analyze differential expression (log2 Fold-Change) between experimental conditions: (1) DNBS vs SHAM, highlighting pathways modulated by the DNBS-induced pro-inflammatory stimulus; (2) DNBS_TAP vs DNBS, showing the impact of TAP on protein profiles after DNBS exposure; and (3) DNBS_TAP vs SHAM, confirming the activation or inhibition of pathways susceptible to TAP’s anti-inflammatory activity. A total of 5451 proteins were identified (Table 1), and the up- and down regulation of proteins was considered statistically significant when log_2_ FC > 0.50 and log_2_ FC < −0.50, respectively (Table S1F, Supporting Information).

**Table 1:**
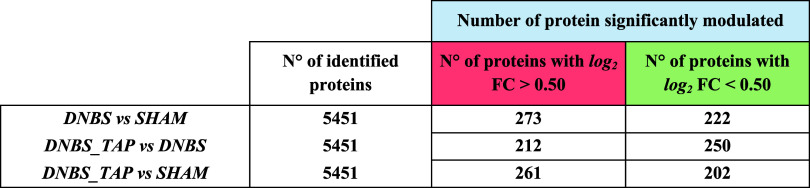
Technical Parameters of Identification and Quantification

The reproducibility of biological replicates (six animals per group) and technical replicates (three independent analyses per biological replicate) was evaluated using Pearson’s linear correlation coefficient across all nLC-HRMS analyses. All correlation coefficients exceeded 0.90, demonstrating strong linear correlation (zoomed multiscatter plot in Figure S1, Supporting Information).

Figure 3 presents Volcano Plots of log2 FC values versus *p*-values, highlighting significantly up-regulated proteins in red and down-regulated proteins in green, with key proteins related to ulcerative colitis mechanisms marked. Panel A shows proteins involved in the inflammatory response, significantly up-regulated in the DNBS vs SHAM comparison. In Panel B (DNBS_TAP vs DNBS), these same proteins exhibit marked down-regulation, indicating the anti-inflammatory effect of the polyphenolic extract. Finally, Panel C (DNBS_TAP vs SHAM) reveals a lower degree of up-regulation compared to Panel A, further supporting the extract’s anti-inflammatory impact.

**Figure 3:**
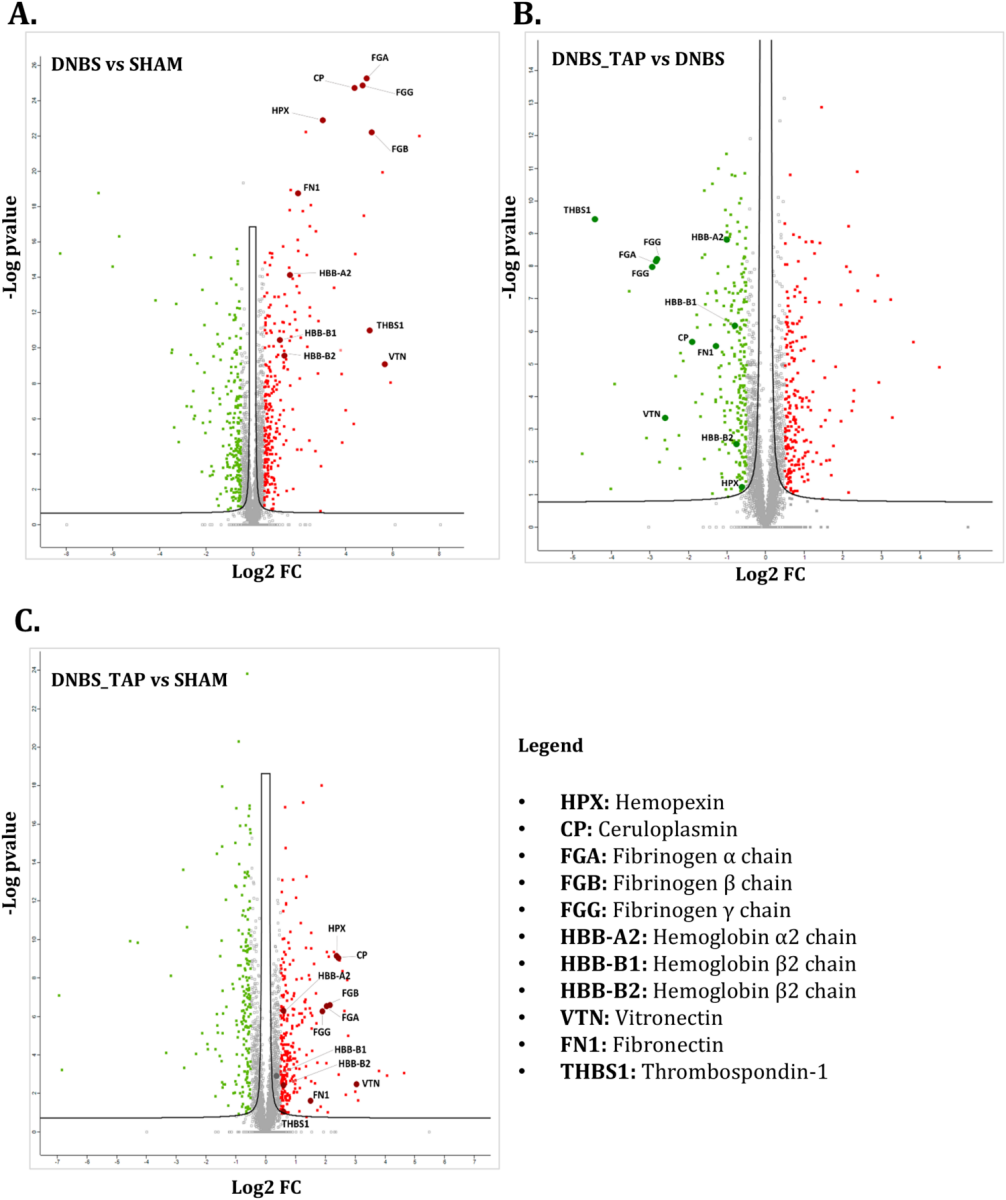
*Volcano Plots* derived from the LFQ analyses of experimental observation 1, DNBS vs SHAM (A), experimental observation 2, DNBS_TAP vs DNBS (B), experimental observation 3 (C), DNBS_TAP vs SHAM.

### 3.3 Pathway and Network Analysis

#### 3.3.1 Identification of Multiprotein Complexes Involved in the Onset of the UC Phenotype (DNBS vs SHAM)

STRING analysis generated protein–protein interaction networks for proteins significantly modulated by DNBS treatment (DNBS vs SHAM). Figure 4 shows the network of up-regulated proteins (log2 FC > 0.50), highlighting key subnetworks involved in immune system activation, oxidative stress response, protection against heme toxicity, induction of ferroptosis, wound healing, and reduction of intestinal mucus layer thickness. The detailed molecular mechanisms within each subnetwork are described in the following paragraphs.

**Figure 4:**
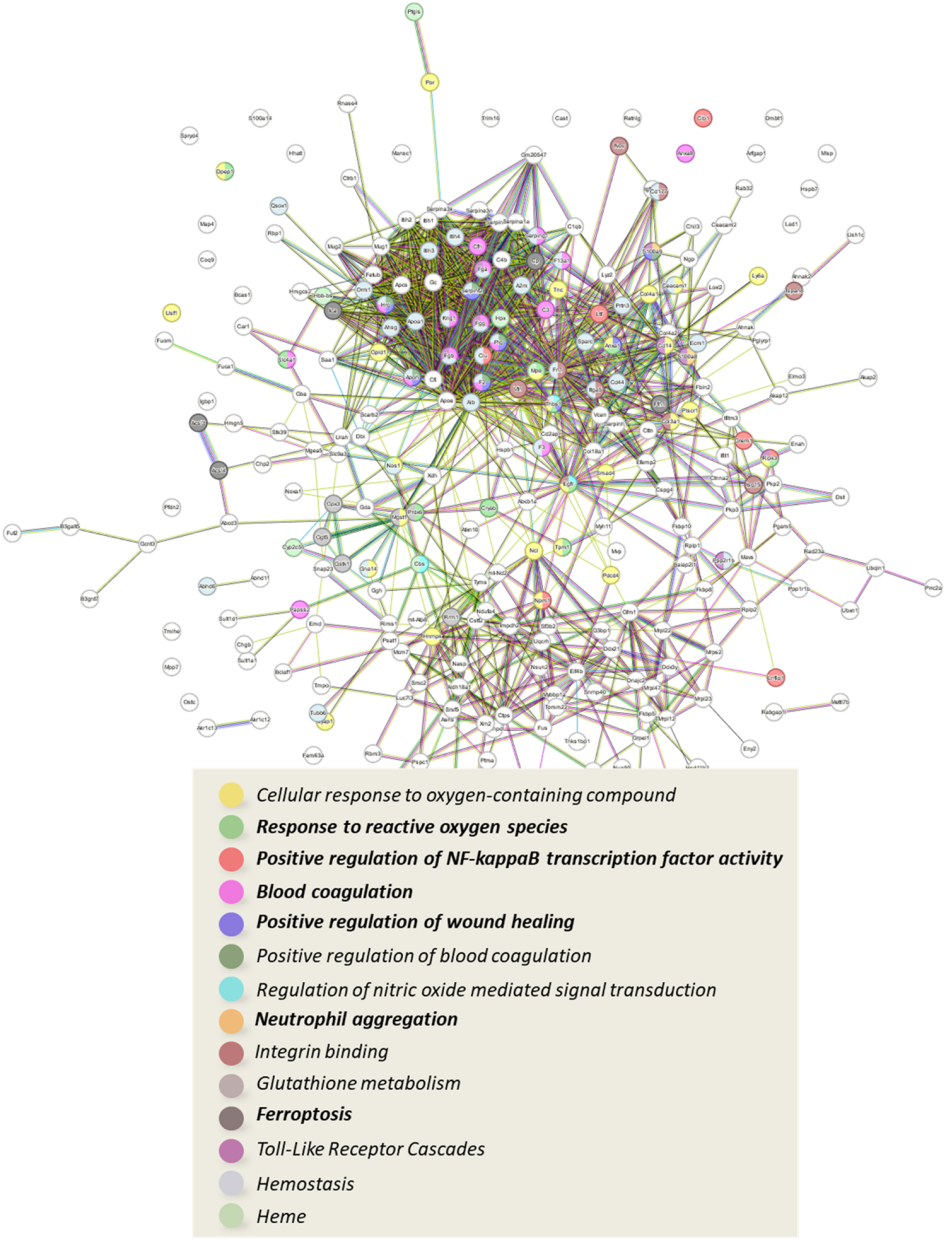
Graphical representation of the network of interactions between proteins that are significantly up-regulated following a pro-inflammatory stimulus (DNBS); below is a list of the most representative biological processes and cellular mechanisms, highlighted by STRING enrichment tool.

##### 3.3.1.1 Activation of the Immune System

In addition to causing direct chemical damage, DNBS acts as a hapten, stimulating the immune response, as shown by STRING analysis (Figure 4), which highlighted the modulation of subnetworks linked to immune activation, such as integrin binding (strength 0.79, FDR 0.0012). Integrins mediate leukocyte adhesion to the basal epithelium, promoting extravasation to damaged sites.^19,20^ IPA also indicated macrophage activation, with up/down-regulated genes (Figure 5, Table S2) linked to cytokine secretion (e.g., TNFα, IL-23), contributing to IBD pathogenesis.^21^

**Figure 5:**
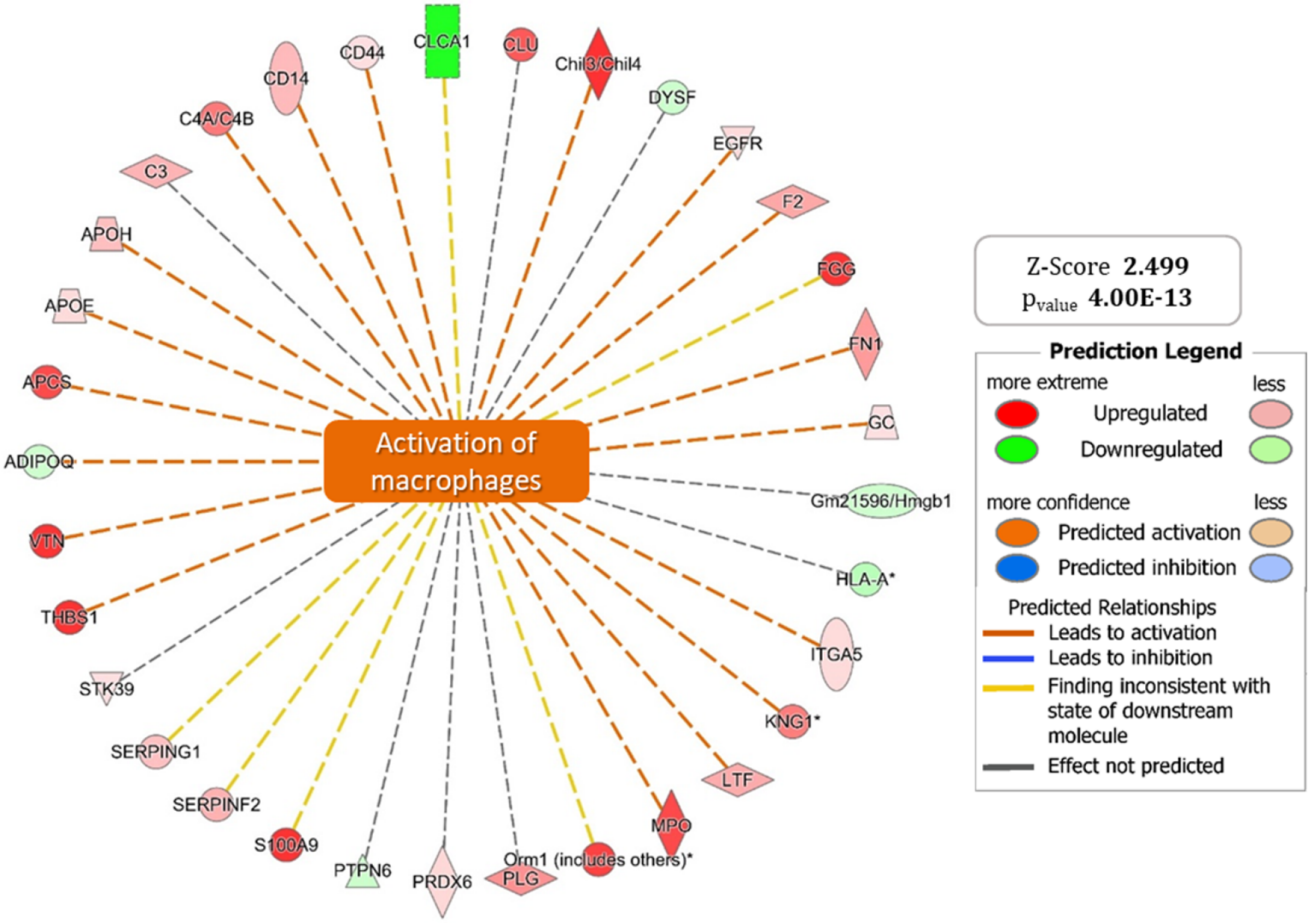
Graphical representation of the “*Activation of macropaghes*” *pathway* for which IPA predicts significant activation with a Z-SCORE value of 2.499. The set of up (red) and down (green) regulated genes supporting the generated hypothesis are shown at the ends of the wheel graph.

IPA analysis also predicts the activation of the “*acute phase signaling*” pathway in DNBS samples (*data not shown*), supported by further predictions of increased levels of interleukin-6 (Figure S2-A, Table S3) and of interleukin-1 (Figure S2–C, Table S4) together with increased expression levels of its receptor IL-6R (Figure S2–B, Table S5) and the prediction of activation of all associated biological processes; this latter information aligns with extensive literature evidence supporting the role of interleukin IL-6 in driving the inflammatory processes characteristic of UC’s pathological profile.^22^ The increase in neutrophil levels is a typical response aimed at eliminating microorganisms or foreign substances by releasing inflammatory mediators. In this context, IPA predicted neutrophil activation (increase in “*Neutrophil quantity*” network, Figure S3, Table S6). This suggests that neutrophil recruitment is triggered by DNBS-induced chemical injury as part of the immune defense mechanism. Confirming the above, through the “upstream regulator” tool in IPA, which aims at identifying the cascade of “upstream” transcriptional regulators that can explain the gene expression changes observed in a “downstream” data set (overexpression of EGFR, FN1, S100A9 and S100A8), an increase in the levels and activation of the transcription factor NF-κB (Nuclear factor kappa B) was suggested, although it could not be detected in MS (Figure S2-D, Table S7). Once activated, NF-κB regulates various cellular processes essential for inflammation and immune response. Prolonged activation has been implicated in many chronic inflammatory diseases, including IBD.^23^

##### 3.3.1.2 Oxidative Stress and Defense Mechanisms

Starting from the network of significantly up-regulated proteins in DNBS-treated samples (DNBS vs SHAM), subnetworks related to oxidative stress were isolated. Up-regulated proteins include myeloperoxidase (MPO), which generates reactive oxygen species and correlates with increased neutrophil levels and MPO activity (Figure 2, panel E). An additional example is given by the upregulation of calprotectin, the heterodimer of the proteins S100A8 and S100A9, which is a ligand of the toll-like receptor 4 (TLR4) and of the receptor for advanced glycation end products (RAGE),^24^ which generates oxidative stress via activation of NADPH oxidase.^25^ Calprotectin overexpression is concordant with colonic inflammation, and, in humans, its quantitative assessment in feces is a useful marker of inflammation in UC.^26^ STRING analysis also highlights several metabolic pathways involved in the elimination of excess reactive oxygen and nitrogen species (ROS and RNS), which are responsible for the oxidative damage including (i) *Hydrogen peroxide catabolic process (strength* 2.64, FDR 0.000085) and (ii) *Response to oxidative stress (strength* 1.54, FDR 0.00017), both with antioxidant functions (Figure S4 – Subnetwork A).

Among the catabolic pathways activated to counteract the excessive production of ROS is the well-defined protein cluster involved in glutathione metabolic processes (Figure S4 – Subnetwork B);^27^ the cluster includes glutathione peroxidase 3 (GPX3), microsomal glutathione S-transferase 1 (MGST1) and γ-glutamyl transferase 5 (GGT5), which in the DNBS vs SHAM comparison matrix have log_2_ FC values of 0.53, 0.57, and 0.54 respectively.

##### 3.3.1.3 Onset of Heme-Mediated Toxicity Mechanism

Colonic bleeding, and an increase in hemoglobin, was observed in DNBS-treated animals compared to SHAM, as indicated by log2 FC values for hemoglobin β chain 1 (1.57), β chain 2 (1.35), and α chain 1 (1.59). STRING analysis highlighted mechanisms linked to ulceration and heme-mediated toxicity. Hemopexin (HPX) was significantly up-regulated (log2 FC 2.98)^28^ due to its detoxifying capacity in binding the heme group. Haptoglobin (HP), which binds free hemoglobin, also showed an increase. Figure 6 shows LFQ intensity values for HPX and HP, along with the effect of TAP treatment described in the following section.

**Figure 6:**
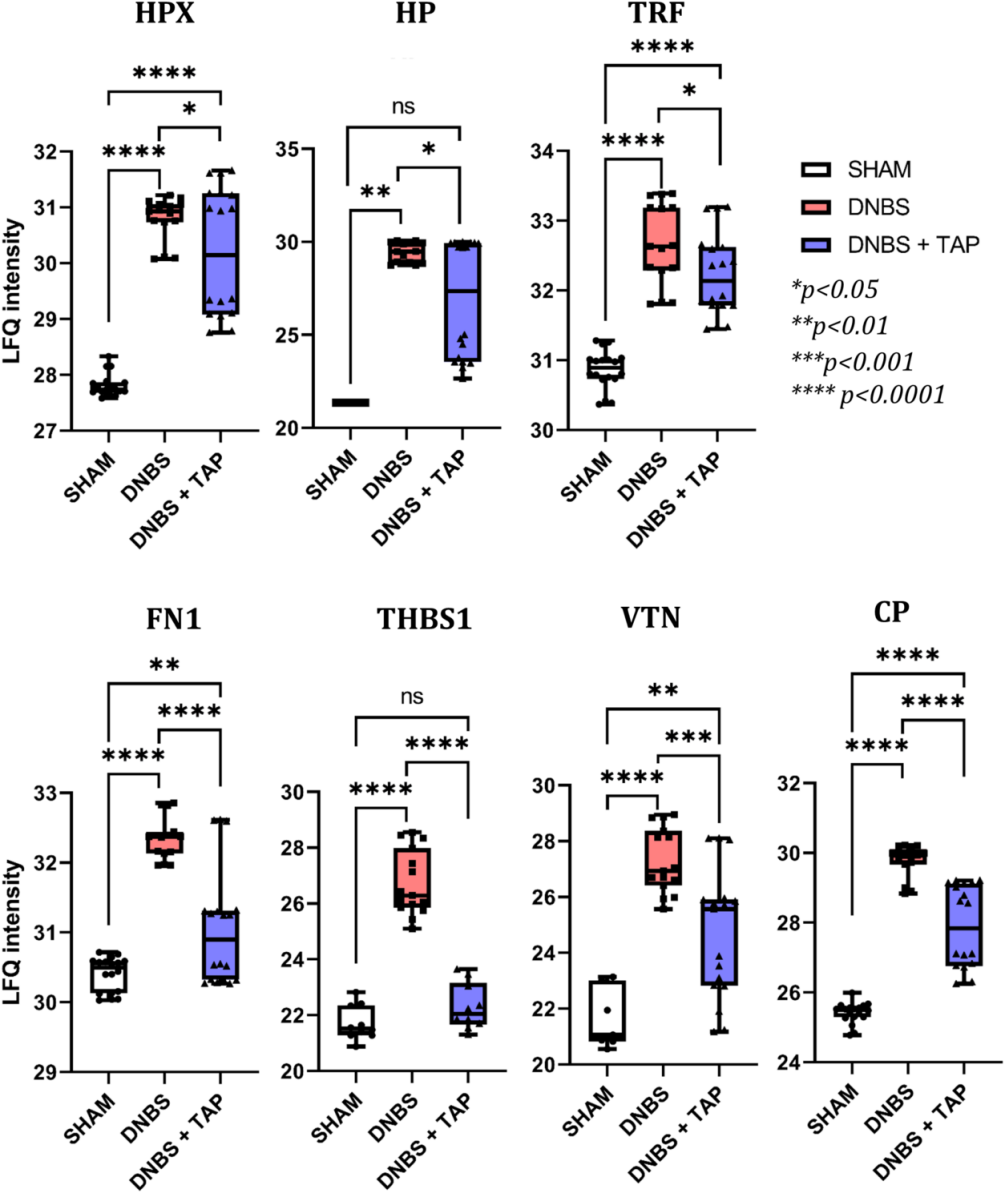
Graphical representation of the distribution of LFQ *intensity* values recorded for hemopexin (HPX), haptoglobin (HP), transferrin (TRF), fibronectin (FN1), thrombospondin (THBS1), vitronectin (VTN) and ceruloplasmin (CP). In all the experimental conditions (SHAM, DNBS, DNBS + TAP).

LFQ intensity values for both proteins were significantly higher in the DNBS condition compared to SHAM, with log2 FC values of 2.98 for hemopexin and 8.05 for haptoglobin. Other significantly regulated proteins involved in defense against heme toxicity include ceruloplasmin (CP), vitronectin (VTN), fibronectin (FN), transferrin (TRF), and thrombospondin (THBS1) (Figure 6). Overall, DNBS-induced ulceration and bleeding elevate hemoglobin levels, leading to heme-related toxicity.^29^ This triggers detoxification processes involving HPX, VTN, CP, and FN, all members of the hemopexin superfamily, known for their roles in leukocyte adhesion, immune response modulation, and coagulation inhibition - hallmarks of the ulcerative inflammatory state.

##### 3.3.1.4 Activation of Ferroptosis

STRING analysis also revealed a subset of up-regulated proteins involved in the cellular pathway of ferroptosis (*strength* 2.74, FDR 1.95 × 10^–14^). Among the proteins involved in ferroptosis (Figure S4 – Subnetwork C), in addition to transferrin (TRF, *log*_2_ *FC* 1.80), ceruloplasmin (CP, *log*_2_ *FC* 1.55) and ferritin 1 heavy chain (FTH1, *log*_2_ *FC* 1.74), already mentioned in the iron homeostasis path (*strength* 2.72, FDR 0.0015), the presence of gene products such as ACSL3 (*log*_2_ *FC* 1.12) and ACSL4 (*log*_2_ *FC* 0.90), the Acid-CoA ligase to long-chain fatty acids 3 and 4, respectively, was noted; these proteins, in addition to being involved in the fatty acid biosynthesis pathway (*strength* 2.59, FDR 0.0014), are crucial for ferroptosis since this kind of nonapoptotic cell death is triggered by the accumulation of membrane lipid peroxides due to iron overload.^30^

##### 3.3.1.5 Activating Wound Healing

In response to DNBS-induced ulcers and hemorrhages, a significant activation of coagulation and wound healing mechanisms was observed. Functional analysis (STRING, Figure 4) revealed an overexpression of proteins linked to hemostasis (*strength* 0.74, FDR 4.16 × 10^–13^) and fibrin clot formation (*strength* 1.77, FDR 2.05 × 10^–05^), alongside proteins involved in inflammation, re-epithelialization, and angiogenesis. Figure S4—Subnetwork D highlights proteins implicated in re-epithelialization and angiogenesis (e.g., tissue factor III, collagen IV α2, thrombospondin 1),^31^ which contribute to tissue regeneration by promoting revascularization and cellular sustenance. IPA analysis also supports these findings, showing downstream inhibition of bleeding processes with a Z-SCORE of −1.837 (Figure 7, Table S8), which can be interpreted as an effect of the activation of key gene products promoting healing in response to tissue damage.

**Figure 7:**
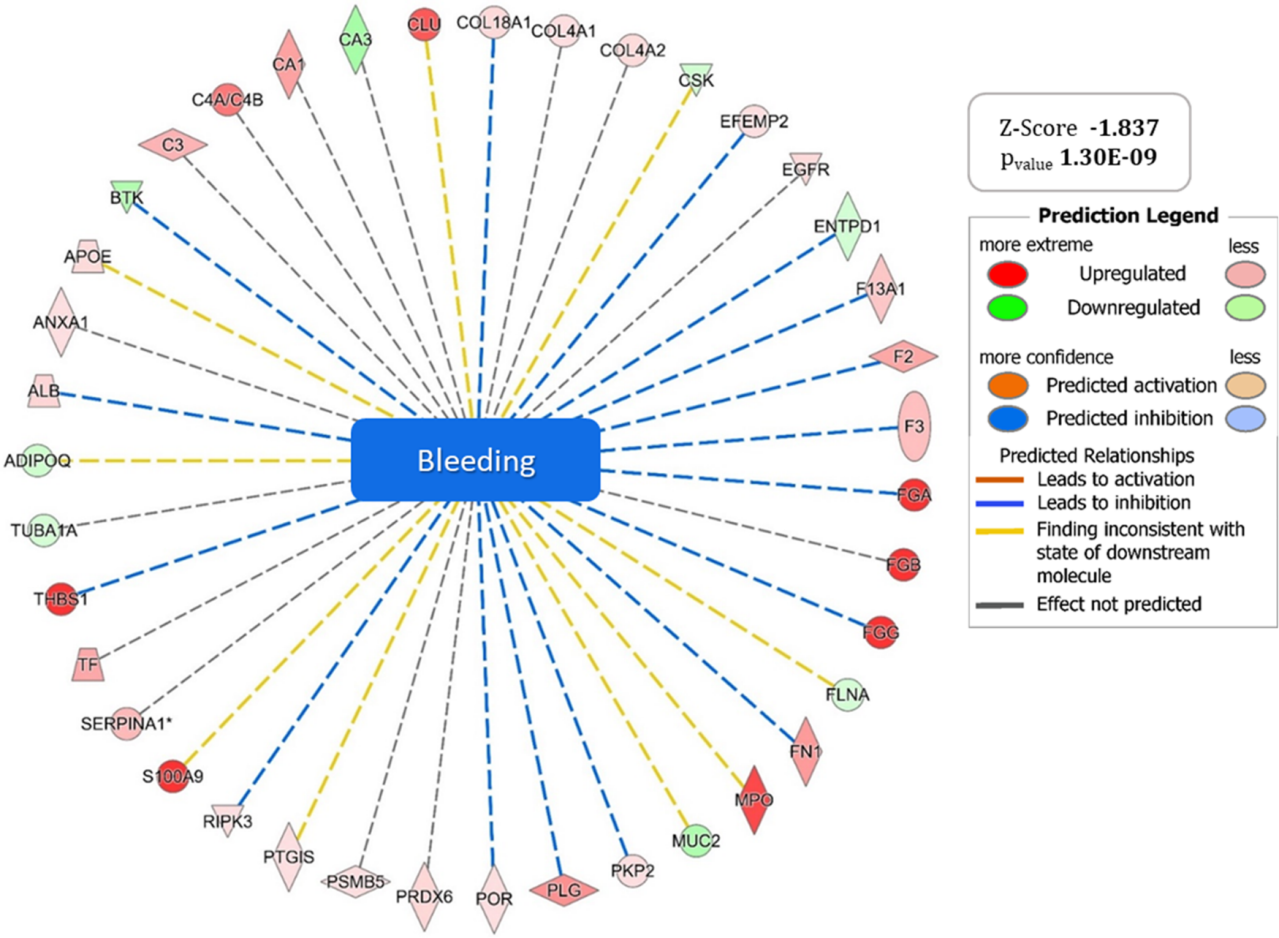
Graphical representation of the “*Bleeding*” pathway for which IPA predicts significant inhibition with a Z-SCORE value of −1.837. The set of up (red) and down (green) regulated genes supporting the generated hypothesis are shown at the ends of the wheel graph.

##### 3.3.1.6 Decreased Thickness of the Mucus Layer of the Intestinal Wall

A reduced expression of the MUC2 gene product, one of the mucus-secreting proteins, was observed in the DNBS vs SHAM comparison matrix data set (*log*_2_ *FC* equal to −0.99). Mucins are glycoproteins produced by epithelial cells and secreted to form the mucus that provides a physiological barrier. Reduction in the thickness of the mucosa covering the epithelium of the organ, which serves to protect it from external damage, certainly contributes to the onset of colon tissue damage.

##### 3.3.1.7 Response to the Inflammatory State

In DNBS-treated animals, IPA predicted an inhibitory effect to the inflammatory process (Z-SCORE −1.698), as an adaptive mechanism in response to the inflammatory process itself. The set of up- and down-regulated genes supporting the generated hypothesis are reported in the Supporting Information (Figure S5, Table S9).

#### 3.3.2 Elucidation of the Molecular Mechanisms Underlying the Anti-Inflammatory Effects Promoted by the Polyphenolic Extract TAP

After elucidating the molecular mechanisms behind DNBS-induced colonic inflammation, attention shifted to assessing the efficacy of TAP extract, administered orally, in mitigating this damage. Quantitative proteomics revealed that TAP successfully reverted the activated inflammatory pathways triggered by DNBS

##### 3.3.2.1 TAP-Mediated Reversal of Mechanisms Activated by DNBS

Of particular interest was the functional investigation of significantly down-regulated proteins in mice exposed to DNBS and treated with the TAP extract (DNBS_TAP vs DNBS), whose network of interactions extracted with the STRING software is shown in Figure 8.

**Figure 8:**
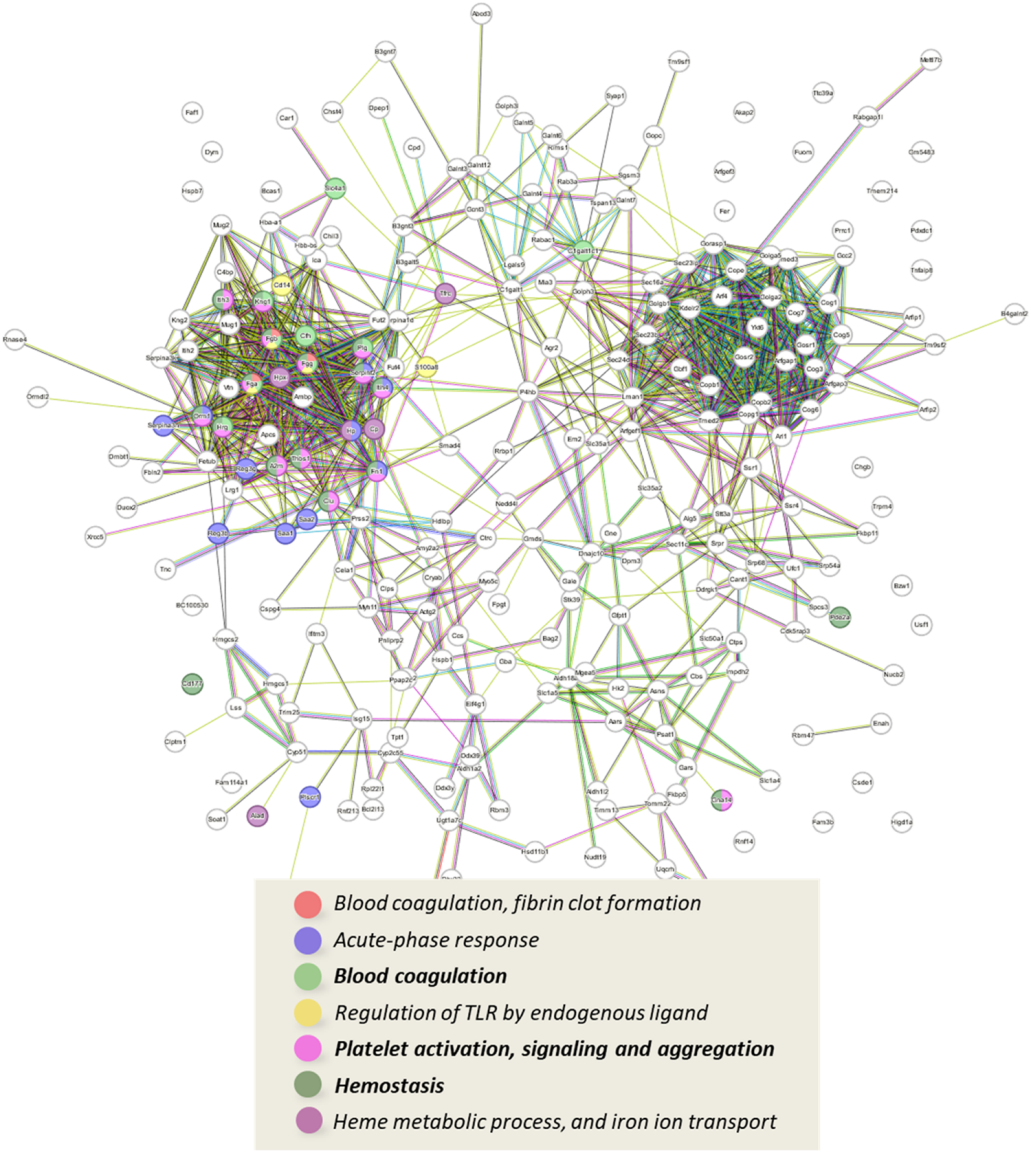
Representation of the network of interactions between significantly down-regulated proteins in the DNBS_TAP vs DNBS comparison matrix; below is a list of the most representative biological processes and cellular mechanisms for the study, highlighted by means of the STRING enrichment tool.

The activation of molecular pathways involved in blood coagulation, hemostasis, and overall wound healing observed in the DNBS vs SHAM comparison was notably reversed following TAP treatment (Table 2), demonstrating its modulatory effect on DNBS-induced damage. This trend reversal was particularly pronounced for heme-toxicity-related proteins, such as HP, HPX and hemoglobin subunits (HBB1, HBB2, HBA2), which showed significantly lower LFQ values post-treatment (Figure 6, Table 2), with levels approaching those seen in the SHAM group.^29^ For wound-healing proteins, there is an overall decrease in expression levels, as reduced tissue ulceration lowers the need to activate healing mechanisms such as coagulation, inflammation, and angiogenesis. Table 2 provides the log2 FC values for key gene products involved in wound healing, including coagulation factors I and II and fibrinogen α-, β-, and γ-chains.

**Table 2:**
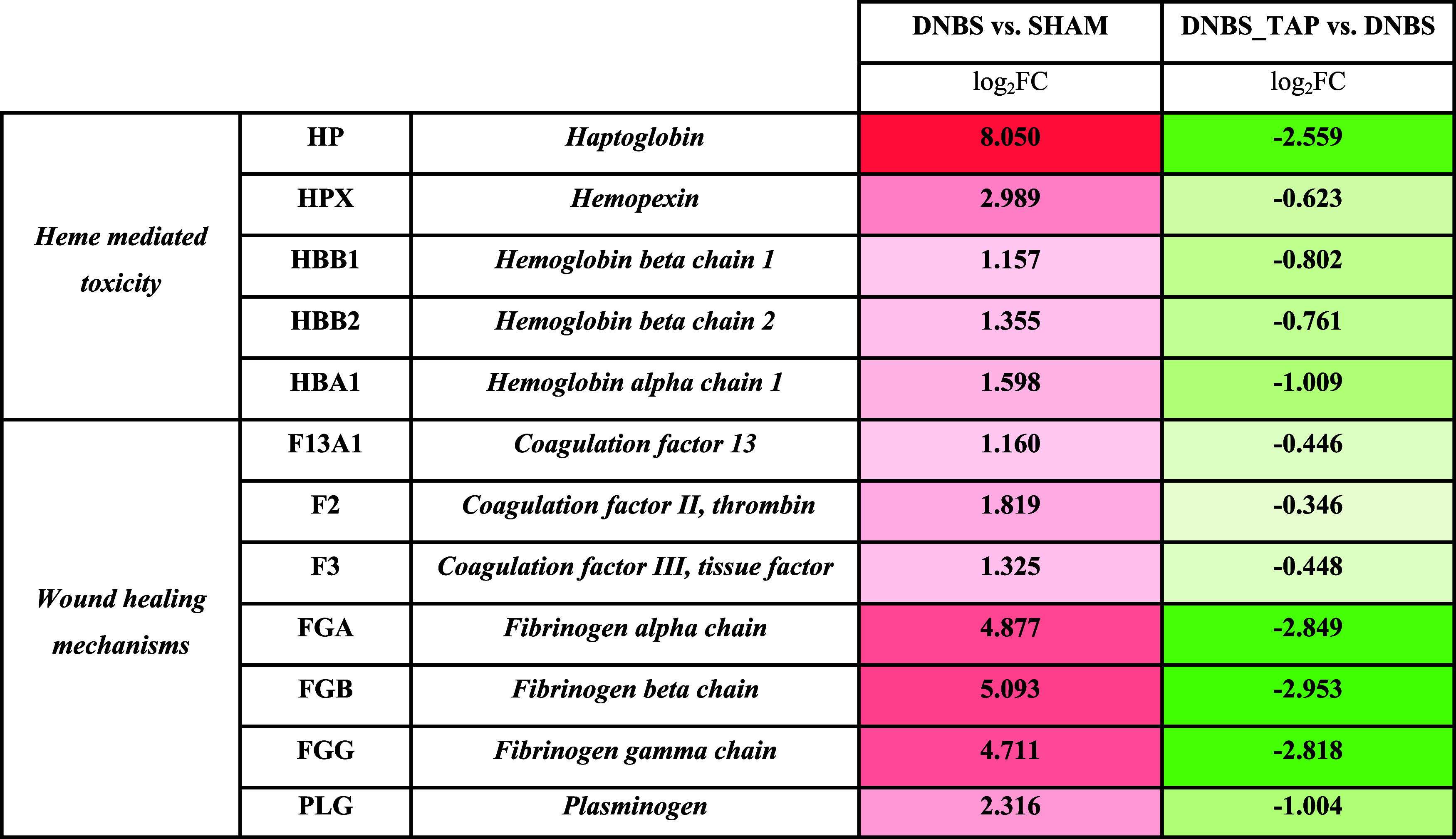
Panel of *log*_2_ *FC* Values Extrapolated from the Three Comparison Matrices for Some of the Proteins Involved in the Mechanisms of Heme-Mediated Toxicity and Wound Healing Mechanisms^a^

a Red indicates up-regulation and green down-regulation, with color intensity reflecting the magnitude of the variation.

The up-regulation of vitronectin, fibronectin, and thrombospondin observed in DNBS-treated animals is significantly reduced following TAP treatment, as shown in Figure 6. This trend is consistent across all proteins, with DNBS causing a notable increase in their levels, which TAP treatment substantially lowers, though not completely to SHAM (physiological) levels.

IPA analyses further corroborate the reversal of processes involved in ulcerative colitis pathogenesis. The comparison between DNBS_TAP and DNBS reveals two key protein clusters: the first includes neutrophil-related proteins (Figure S6, Table S10), whose inhibition aligns with reduced immune cell recruitment due to TAP’s anti-inflammatory effects. The second cluster pertains to the “*Acute Phase Signaling*” pathway, where IPA predicts a significant inhibition (Z-SCORE −2.00) following TAP treatment, indicating a reduced activation of the immune system (Figure 9, Table S11). The network also suggests the inhibition of NF-κB and IL-6, both of which play critical roles in the development of chronic intestinal inflammation, as previously discussed.

**Figure 9:**
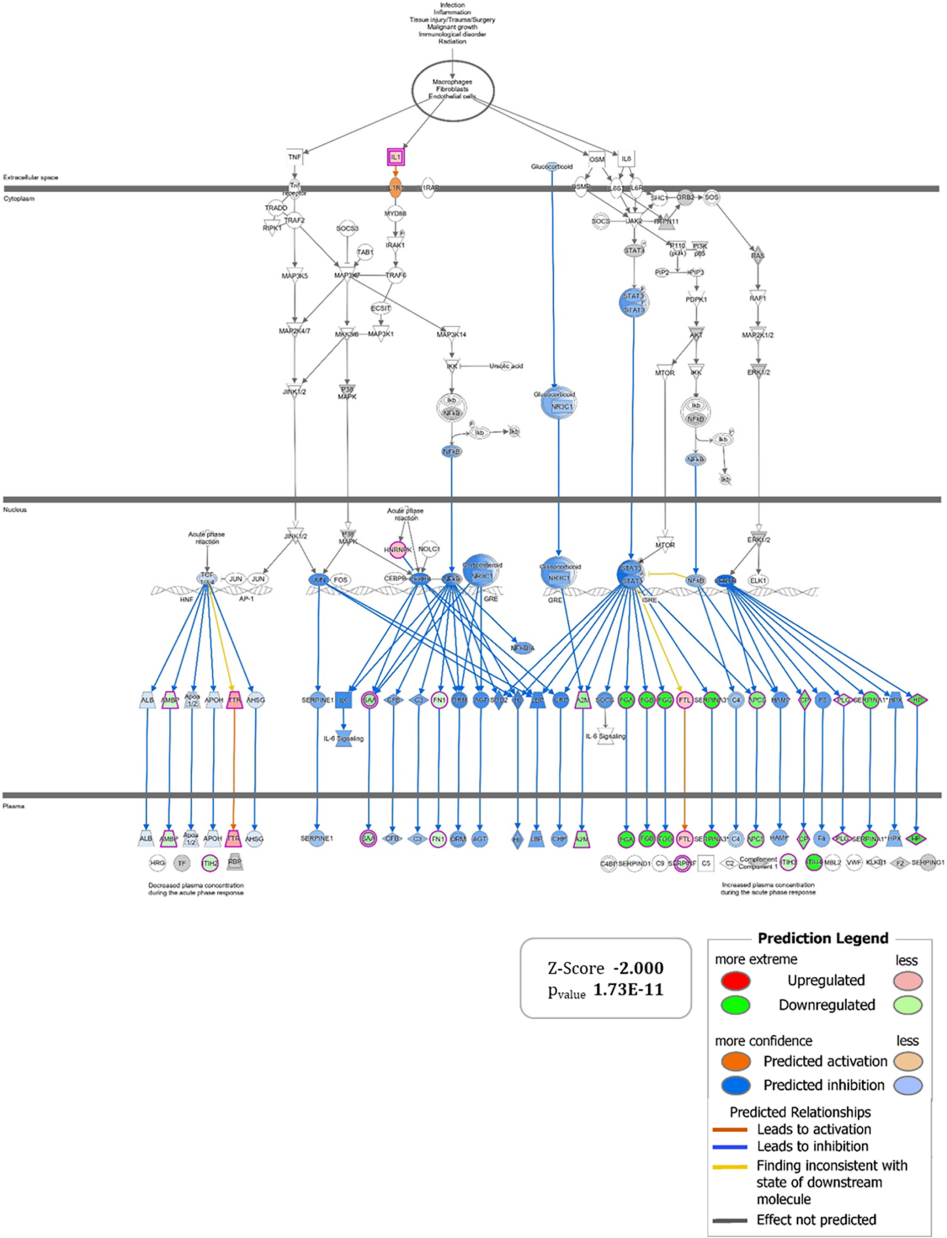
Graphical representation of the “*Acute phase signaling*” *pathway’* for which IPA predicts significant inhibition with a Z-SCORE value of −1.698. The set of genes *up* (red) and *down* (green) regulated to support the hypothesis generated are shown at the ends of the wheel graph.

The interpretation of the log2 FC values referred to the DNBS_TAP vs DNBS comparison suggests inhibition of the NOS2 (*Nitric Oxide Synthase 2*) mechanism, as indicated by the IPA upstream regulator function (data not shown). NOS2, the inducible form of nitric oxide synthase, is typically expressed in response to stimuli like LPS or IFN-γ. Its levels were substantially increased in the DNBS vs SHAM comparison (Z-SCORE 3.63; *p*-value 2.64 × 10^–09^), promoting inflammation and favoring the formation of reactive nitrogen species (RNS).^32,33^ Following TAP treatment, NOS2 levels drastically decreased (Z-SCORE −2.52; *p*-value 4.22 × 10^–09^), suggesting reduced nitric oxide production and an improvement in the inflammatory condition, with values approaching the physiological state (DNBS_TAP vs SHAM).

##### 3.3.2.2 TAP Induces an Antioxidant Response

The down-regulation of proteins in TAP-treated samples, along with morphological and histological assessments, confirms TAP’s effectiveness in reducing DNBS-induced inflammation. However, the exact molecular mechanism remains unclear, likely involving the interlinked processes of oxidative stress and inflammation.^34^ A common approach to disrupting chronic inflammation is through inhibiting oxidative stress by activating Nrf2, a mechanism previously demonstrated by TAP *in vitro*, showing dose-dependent anti-inflammatory effect.^17^ TAP treatment here promoted an antioxidant response by inducing Nrf2-regulated detoxifying/antioxidant enzymes, including SOD1 and SOD3, which convert superoxide anions into oxygen and hydrogen peroxide, thereby mitigating free radical damage. It also increased levels of glutathione peroxidase 1 (GPX1), which reduces hydrogen peroxide using GSH as a cofactor, NAD­(P)H dehydrogenase (quinone 1)-NQO1, which detoxifies quinones through an NAD­(P)­H-mediated reaction,^35^ and peroxiredoxin 6 (PRDX6), a nonselenic peroxidase. The relative enzyme levels across the three experimental conditions are shown in Figure 10.

**Figure 10:**
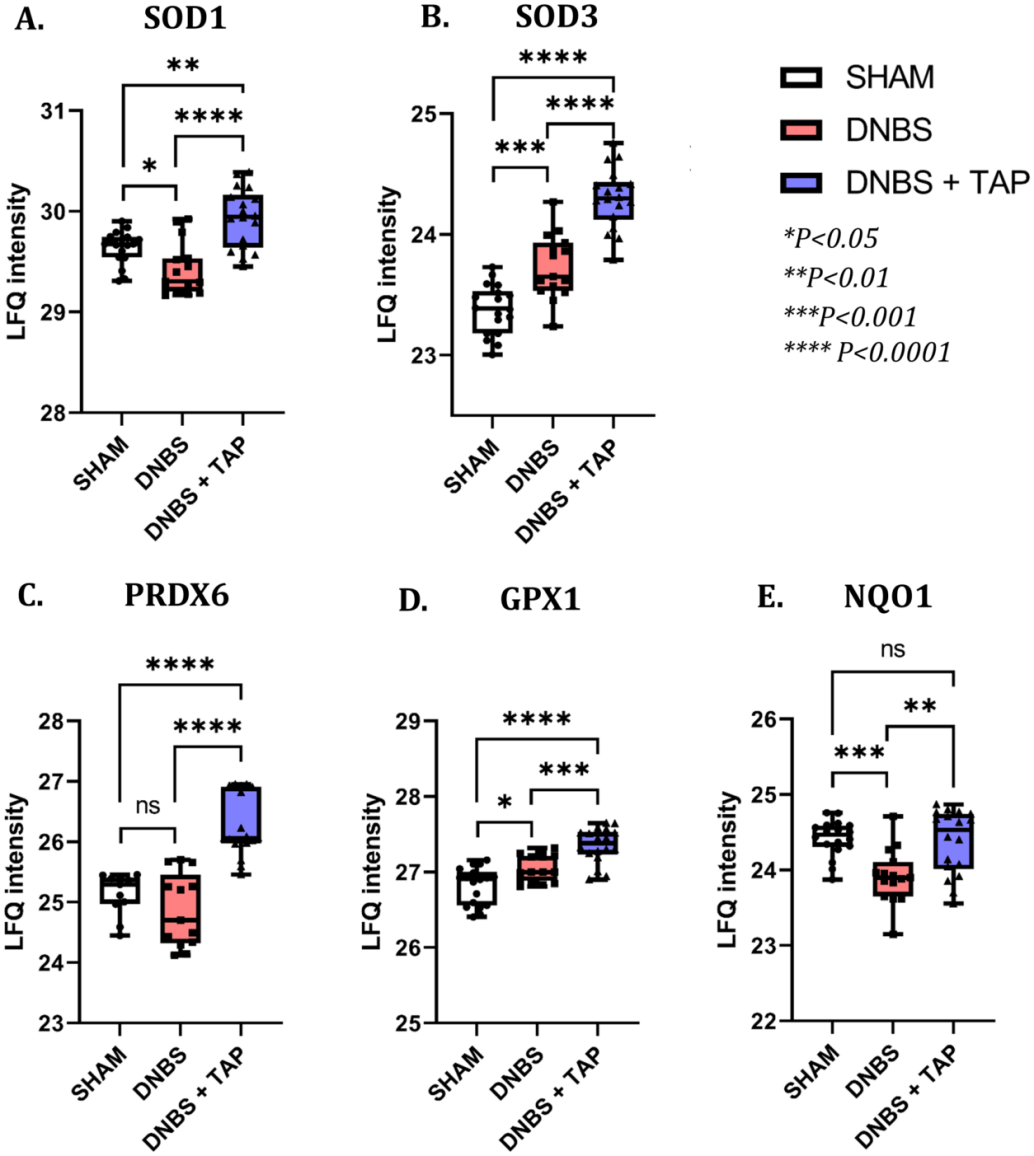
Graphical representation of the distribution of LFQ *intensity* values recorded for Superoxide dismutase 1 and 3 (SOD1, SOD3), glutathione peroxidase 1 (GPX1), NAD­(P)H dehydrogenase (quinone 1) (NQO1), and peroxiredoxin 6 (PRDX6) in all the experimental conditions (SHAM, DNBS, DNBS + TAP).

It is worth highlighting that SOD1 was down-regulated in the DNBS group while SOD3 was up-regulated. Previous studies have shown that SOD3, but not SOD1 (or SOD2), is regulated through Nrf2 ^36^ and this result would explain the expression of SOD3 in DNBS treated animals, a sign of activation of the first line of defense against oxidative stress/inflammation through the Keap1-Nrf2 signaling pathway; on the contrary, SOD1 was not activated but even reduced. As for SOD1, the relative contents of peroxiredoxin 6 (PRDX6) and NQO1 in DNBS treated animals were not stimulated by the pro-inflammatory stimulus. TAP not only enhanced the expression of antioxidant enzymes unaffected by inflammation, such as SOD1, PRDX6, and NQO1, but also increased those activated by DNBS, such as SOD3 and GPX1, suggesting both Nrf2-dependent and independent antioxidant activation mechanisms.

Notably, in physiological conditions TAP did not impact the Nrf2 response thus demonstrating that it operates only in the presence of an oxidative stress stimulus. It is tempting to speculate that the presence of an ortho-diphenol moiety in some compounds may underlie their role as TAP constituents potentially contributing to Nrf2 activation.^18^ These compounds could act as electrophilic binder of the thiol group of Keap1 when activated as quinones and such an activation is mediated by iron and peroxides which are activated in inflammatory condition such as that induced by DNBS.

Finally, among the most significant *subnetworks*, protein *clusters* involved in the neutralization of toxic products for the cell are well recognized, which are essential for restoring the redox balance in inflamed tissue (*Antioxidant activity*, *strength* 2.5, FDR 1.28 × 10^–09^) (Figure S7 – Subnetwork E).

### 3.4 Targeted ELISA Validation of Haptoglobin and Ceruloplasmin

Based on the proteomic analysis results and the potential functional significance of the identified proteins, we selected two candidates for further validation: haptoglobin and ceruloplasmin. To confirm their expression levels in our samples, colon homogenates were pretreated to remove any residual detergent from the lysis buffer before performing the ELISAs. Each sample was then assayed in technical triplicate at two dilutions (1:100 and 1:200 in the kit’s assay diluent) to ensure that the readings fell within the standard calibration curve.

The ELISA results indicated that both haptoglobin and ceruloplasmin levels were elevated in the DNBS colitis group compared to the control (Sham) group. Specifically, haptoglobin was undetectable in Sham samples, whereas it reached a median of 1.0169 μg/mL (range 0.4949–1.6348) in the DNBS group, then decreased to 0.3087 μg/mL (0–1.4062) in the DNBS_TAP group. Similarly, ceruloplasmin levels in Sham animals were 153.2310 μg/mL (143.4580–171.2078), which increased to 189.2043 μg/mL (156.7479–214.0201) in DNBS colitis and dropped to 136.1409 μg/mL (128.3955–144.3267) in the DNBS_TAP group. Although both proteins showed a significant increase in the DNBS group compared to SHAM controls, and a significant reduction following TAP treatment compared to DNBS alone, no statistically significant differences were observed between the DNBS_TAP group and SHAM. This pattern indicates that TAP administration effectively restored protein levels toward baseline values (Figure 11, panel A and B).

**Figure 11:**
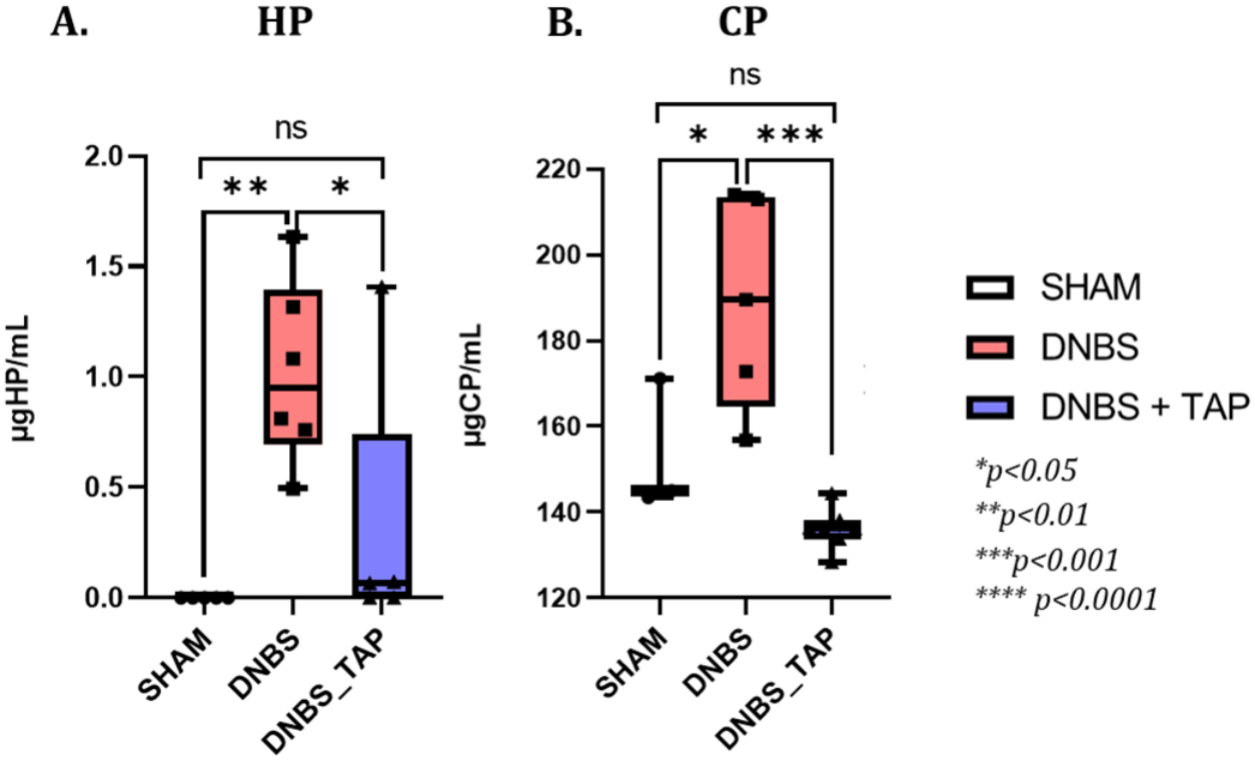
Distribution of the measured concentrations (μg/mL) of haptoglobin (HP) and ceruloplasmin (CP) quantified by ELISA across all experimental groups, including untreated controls (CTRL), DNBS-induced colitis (DNBS), and DNBS animals treated with thinned apple polyphenol extract (DNBS + TAP).

## 4 Conclusions

In conclusion, this study demonstrates the significant anti-inflammatory potential of thinned apple polyphenols (TAP) in a DNBS-induced colitis mouse model. TAP treatment effectively modulated key molecular pathways disrupted by colonic inflammation, including those related to ferroptosis, heme toxicity, and oxidative stress, while possibly fostering an antioxidant response through Nrf2 activation. Importantly, TAP was able to revert the expression levels of numerous inflammatory mediators and restore physiological protein profiles, indicating a strong therapeutic effect on tissue regeneration and healing. These findings suggest that TAP could serve as a promising candidate for developing innovative therapeutic strategies targeting chronic intestinal inflammation. Moreover, utilizing TAP aligns with sustainable development goals by valorizing agricultural byproducts, offering a dual benefit of health improvement and waste reduction. Such an approach underscores the potential of TAP as a cost-effective, natural source of bioactive compounds for future applications in the treatment of inflammatory bowel diseases.

## Supplementary Material





## Data Availability

The data sets generated during and/or analyzed during the current study are available. Specifically, the mass spectrometry proteomics data have been deposited in the PRIDE partner repository for the ProteomeXchange Consortium with PRIDE accession number PXD056914.

## Notes

**Figure S1** - Evaluation of the linear correlation of LFQ intensity values between biological and technical replicates by calculating Pearson’s linear correlation coefficient. **Table S2** - Proteins contributing to the predicted activation of the “Activation of macrophages” function, as identified by Ingenuity Pathway Analysis (IPA) in the Diseases and Functions category. The table lists, for each protein, its expression log ratio, direction of regulation, and related biological findings. Proteins displayed inFigure 5 correspond to those included in this analysis. **Figure S2** - Graphical representation of the subset of proteins that, based on their modulation (up/down-regulation), support the activation hypothesis of IL-6 (A) and its receptor IL-6R (B) in the DNBS vs SHAM comparison matrix. In panel C of the subset of proteins that support the hypothesis of IL-1 activation, while in panel D of NF-kB pathway. **Table S3** - Proteins contributing to the predicted activation of the upstream regulator IL-6, as identified by Ingenuity Pathway Analysis (IPA). The table reports, for each protein, gene symbol, expression value (log ratio and *p*-value), subcellular localization, and functional family. Proteins shown correspond to those supporting the activation of IL-6 reported in Figure S2_A. **Table S4** - Proteins contributing to the predicted activation of the upstream regulator IL-1, as identified by Ingenuity Pathway Analysis (IPA). The table reports, for each protein, gene symbol, expression value (log ratio and *p*-value), subcellular localization, and functional family. Proteins shown correspond to those supporting the activation of IL-1 reported in Figure S2_C. **Table S5 -** Proteins contributing to the predicted activation of the upstream regulator IL6R, as identified by Ingenuity Pathway Analysis (IPA). The table reports, for each protein, gene symbol, expression value (log ratio and *p*-value), subcellular localization, and functional family. Proteins shown correspond to those supporting the activation of IL6R reported in Figure S2_B. **Figure S3** - Graphical representation of the “Quantity of neutrophils” pathway for which IPA predicts significant activation with a Z-SCORE value of 2.476. The set of genes up (red) and down (green) regulated to support the generated hypothesis are shown at the ends of the wheel graph. **Table S6** - Proteins contributing to the predicted activation of the “Quantity of neutrophils” function, as identified by Ingenuity Pathway Analysis (IPA) in the Diseases and Functions category. The table lists, for each protein, its expression log ratio, direction of regulation, and related biological findings. Proteins displayed in Figure S3 correspond to those included in this analysis. **Table S7** - Proteins contributing to the predicted activation of the upstream regulator NFKB, as identified by Ingenuity Pathway Analysis (IPA). The table reports, for each protein, gene symbol, expression value (log ratio and *p*-value), subcellular localization, and functional family. Proteins shown correspond to those supporting the activation of NFKB reported in Figure S2_D. **Figure S4** - List of subnetworks of significantly up-regulated proteins (DNBS vs SHAM comparison matrix), united by their involvement in specific biological processes; below the list of protein involved is the color code that associates each gene product with the GO (gene ontology annotation) used for functional enrichment. **Table S8** - Proteins contributing to the predicted activation of the “Bleeding” function, as identified by Ingenuity Pathway Analysis (IPA) in the Diseases and Functions category. The table lists, for each protein, its expression log ratio, direction of regulation, and related biological findings. Proteins displayed inFigure 7 correspond to those included in this analysis. **Figure S5** - Graphical representation of the “Inflammation of organ” pathway for which IPA predicts significant inhibition with a Z-SCORE value of −1.698. The set of genes up (red) and down (green) regulated to support the generated hypothesis are shown at the ends of the wheel graph. **Table S9** - Proteins contributing to the predicted activation of the “Inflammation of organ” function, as identified by Ingenuity Pathway Analysis (IPA) in the Diseases and Functions category. The table lists, for each protein, its expression log ratio, direction of regulation, and related biological findings. Proteins displayed in Figure S5 correspond to those included in this analysis. **Figure S6** - Graphical representation of the “Immune response of neutrophils” pathway for which IPA predicts significant inhibition with a Z-SCORE value of −1.698. The set of genes up (red) and down (green) regulated to support the generated hypothesis are shown at the ends of the wheel graph. **Table S10 -** Proteins contributing to the predicted activation of the “Immune response of neutrophils” function, as identified by Ingenuity Pathway Analysis (IPA) in the Diseases and Functions category. The table lists, for each protein, its expression log ratio, direction of regulation, and related biological findings. Proteins displayed in Figure S6 correspond to those included in this analysis. **Table S11** - Proteins contributing to the predicted activation of the Canonical Pathway identified as “Acute Phese Signaling” by Ingenuity Pathway Analysis (IPA). The table reports, for each protein, gene symbol, expression value (log ratio and *p*-value), subcellular localization, functional family and biomarker application(s). Proteins shown correspond to those supporting the activation of “Acute Phese Signaling” reported in Figure 9. **Figure S7** - List of subnetworks of significantly up-regulated proteins (DNBS_TAP vs DNBS comparison matrix), united by their involvement in specific biological processes; below the list of protein involved is the color code that associates each gene product with the GO (gene ontology annotation) used for functional enrichment (PDF)
**Table S1**. Comprehensive list of proteins significantly modulated in at least one of the three pairwise comparisons: DNBS vs CTR, DNBS_TAP vs DNBS, and DNBS_TAP vs CTR. Proteins were selected based on their statistical significance within each comparison and subsequently merged into a unified matrix using an *in-house* script specifically designed to ensure a consistent cross-condition comparison. It should be noted that not all proteins listed are significantly modulated across all three contrasts; rather, the resulting matrix represents the union of all proteins displaying significant modulation in at least one comparison. This integrative approach was implemented to obtain a complete and harmonized data set suitable for downstream analyses in *Ingenuity Pathway Analysis (IPA)*, including *Biomarker Comparison* and multicondition enrichment studies. For each protein, identified by its gene name, both the *p-value* and *log*_*2*_ *ratio (fold-change)* are reported for the three comparisons. Color coding has been applied to facilitate visual interpretation: red shading denotes proteins showing biologically relevant regulation (|log_2_ ratio| > 0.5), with upregulated proteins in red and downregulated proteins in green. Within the *p*-value columns, nonsignificant values (*p* > 0.05) are also highlighted in red to enable rapid distinction of statistically robust variations (XLSX)

## Abbreviations

ACN: acetonitrile
ARE: antioxidant response element
DNBS: dinitrobenzenesulfonic acid
EGFR: epidermal growth factor receptor
FA: formic acid
FN: fibronectin
FTH: ferritin heavy chain
GPX: glutathione peroxidase
HP: haptoglobin
HPX: hemopexin
IBD: inflammatory bowel disease
IL-1: interleukin-1
IL-6: interleukin-6
LFQ: label-free quantification
MPO: myeloperoxidase
NF-κB: nuclear factor kappa B
NOS: nitric oxide synthase
NSAIDs: non-steroidal anti-inflammatory drugs
Nrf2: nuclear factor erythroid 2–related factor 2
PRDX: peroxiredoxin
RAGE: receptor for advanced glycation end products
SOD: superoxide dismutase
TAP: thinned apple polyphenols
TEAB: triethylammonium bromide
TLR4: toll-like receptor 4
TNF-α: tumor necrosis factor-α
TRF: transferrin
UC: ulcerative colitis
VTN: vitronectin

## References

[1] Crocetti E., Bergamaschi W., Russo A. G. (2021). Population-Based Incidence and Prevalence
of Inflammatory Bowel Diseases in Milan (Northern Italy), and Estimates
for Italy. Eur. J. Gastroenterol. Hepatol..

[2] Aslam N., Lo S. W., Sikafi R., Barnes T., Segal J., Smith P. J., Limdi J. K. (2022). A Review of the Therapeutic Management
of Ulcerative Colitis. Ther. Adv. Gastroenterol..

[3] Zeng Z., Lin H., Jiang M., Yuan J., Li X., Jia Y., Yang L., Zhang H. (2024). Anti-TNFα in Inflammatory Bowel
Disease: From Originators to Biosimilars. Front.
Pharmacol..

[4] Outtier A., Janssens R., Barbier L., Sabino J., Verstockt B., Vermeire S., Huys I., Ferrante M. (2025). Evolution
of Eligibility
Criteria in Inflammatory Bowel Disease Clinical Trials: A Clinical
Trial Databank Analysis. United Eur. Gastroenterol.
J..

[5] Burisch J., Claytor J., Hernandez I., Hou J. K., Kaplan G. G. (2025). The Cost
of Inflammatory Bowel Disease Care: How to Make It Sustainable. Clin. Gastroenterol. Hepatol..

[6] Sendani A. A., Farmani M., Kazemifard N., Ghavami S. B., Sadeghi A. (2024). Molecular
Mechanisms and Therapeutic Effects of Natural Products in Inflammatory
Bowel Disease. Clin. Nutr. Open Sci..

[7] Subudhi R. N., Poonia N., Singh D., Arora V. (2024). Natural Approaches
for the Management of Ulcerative Colitis: Evidence of Preclinical
and Clinical Investigations. Nat. Prod. Bioprospect..

[8] Blagov A. V., Orekhova V. A., Sukhorukov V. N., Melnichenko A. A., Orekhov A. N. (2023). Potential Use of Antioxidant Compounds for the Treatment
of Inflammatory Bowel Disease. Pharmaceuticals.

[9] Tanigawa S., Fujii M., Hou D. (2007). Action of
Nrf2 and Keap1 in ARE-Mediated
NQO1 Expression by Quercetin. Free Radical Biol.
Med..

[10] Qin S., Hou D. (2016). Multiple Regulations
of Keap1/Nrf2 System by Dietary Phytochemicals. Mol. Nutr. Food Res..

[11] Atreya I., Atreya R., Neurath M. F. (2008). NF-κB
in Inflammatory Bowel
Disease. J. Int. Med..

[12] Gado F., Ferrario G., Della Vedova L., Zoanni B., Altomare A., Carini M., Aldini G., D’Amato A., Baron G. (2023). Targeting Nrf2 and NF-κB Signaling
Pathways in Cancer Prevention:
The Role of Apple Phytochemicals. Molecules.

[13] Siracusa R., Fusco R., Peritore A., Cordaro M., D’Amico R., Genovese T., Gugliandolo E., Crupi R., Smeriglio A., Mandalari G., Cuzzocrea S., Di Paola R., Impellizzeri D. (2020). The Antioxidant
and Anti-Inflammatory Properties of Anacardium Occidentale L. Cashew
Nuts in a Mouse Model of Colitis. Nutrients.

[14] Impellizzeri D., Siracusa R., Cordaro M., Peritore A. F., Gugliandolo E., Mancuso G., Midiri A., Di Paola R., Cuzzocrea S. (2018). Therapeutic
Potential of Dinitrobenzene Sulfonic Acid (DNBS)-Induc*ed Coli*tis in Mice by Targeting IL-1β and IL-18. Biochem. Pharmacol..

[15] Impellizzeri D., Bruschetta G., Di Paola R., Ahmad A., Campolo M., Cuzzocrea S., Esposito E., Navarra M. (2015). The Anti-Inflammatory
and Antioxidant Effects of Bergamot Juice Extract (BJe) in an Experimental
Model of Inflammatory Bowel Disease. Clin. Nutr..

[16] Manoni M., Altomare A., Nonnis S., Ferrario G., Mazzoleni S., Tretola M., Bee G., Tedeschi G., Aldini G., Pinotti L. (2024). Preliminary Investigation on the Impact of Salty and
Sugary Former Foods on Pig Liver and Plasma Profiles Using OMICS Approaches. Sci. Rep..

[17] Ferrario G., Baron G., Gado F., Della Vedova L., Bombardelli E., Carini M., D’Amato A., Aldini G., Altomare A. (2022). Polyphenols from Thinned Young Apples:
HPLC-HRMS Profile and Evaluation of Their Anti-Oxidant and Anti-Inflammatory
Activities by Proteomic Studies. Antioxidants.

[18] Baron G., Altomare A., Della Vedova L., Gado F., Quagliano O., Casati S., Tosi N., Bresciani L., Del Rio D., Roda G., D’Amato A., Lammi C., Macorano A., Vittorio S., Vistoli G., Fumagalli L., Carini M., Leone A., Marino M., Del Bo’ C., Miotto G., Ursini F., Morazzoni P., Aldini G. (2024). Unraveling the Parahormetic Mechanism Underlying the
Health-Protecting Effects of Grapeseed Procyanidins. Redox Biol..

[19] Campbell I. D., Humphries M. J. (2011). Integrin Structure, Activation, and
Interactions. Cold Spring Harbor Perspect. Biol..

[20] De
Franceschi N., Hamidi H., Alanko J., Sahgal P., Ivaska J. (2015). Integrin Traffic – the Update. J. Cell Sci..

[21] Na Y. R., Stakenborg M., Seok S. H., Matteoli G. (2019). Macrophages in Intestinal
Inflammation and Resolution: A Potential Therapeutic Target in IBD. Nat. Rev. Gastroenterol. Hepatol..

[22] Shahini A., Shahini A. (2023). Role of Interleukin-6-Mediated
Inflammation in the
Pathogenesis of Inflammatory Bowel Disease: Focus on the Available
Therapeutic Approaches and Gut Microbiome. J.
Cell Commun. Signaling.

[23] Oeckinghaus A., Ghosh S. (2009). The NF- B Family of
Transcription Factors and Its Regulation. Cold
Spring Harbor Perspect. Biol..

[24] Rochette L., Dogon G., Rigal E., Zeller M., Cottin Y., Vergely C. (2022). Involvement of Oxidative
Stress in Protective Cardiac
Functions of Calprotectin. Cells.

[25] Daffu G., del Pozo C. H., O’Shea K. M., Ananthakrishnan R., Ramasamy R., Schmidt A. M. (2013). Radical Roles for
RAGE in the Pathogenesis
of Oxidative Stress in Cardiovascular Diseases and Beyond. Int. J. Mol. Sci..

[26] Gheibi N., Ghorbani M., Shariatifar H., Farasat A. (2019). In Silico Assessment
of Human Calprotectin Subunits (S100A8/A9) in Presence of Sodium and
Calcium Ions Using Molecular Dynamics Simulation Approach. PLoS One.

[27] Diaz-Vivancos P., De Simone A., Kiddle G., Foyer C. H. (2015). Glutathione –
Linking Cell Proliferation to Oxidative Stress. Free Radical Biol. Med..

[28] Piccard H., Van Den Steen P. E., Opdenakker G. (2007). Hemopexin Domains as Multifunctional
Liganding Modules in Matrix Metalloproteinases and Other Proteins. J. Leukocyte Biol..

[29] Montecinos L., Eskew J. D., Smith A. (2019). What Is Next
in This “Age”
of Heme-Driven Pathology and Protection by Hemopexin? An Update and
Links with Iron. Pharmaceuticals.

[30] Yang Y., Zhu T., Wang X., Xiong F., Hu Z., Qiao X., Yuan X., Wang D. (2022). ACSL3 and ACSL4, Distinct Roles in
Ferroptosis and Cancers. Cancers.

[31] Kyriakides T. R., MacLauchlan S. (2009). The Role of
Thrombospondins in Wound Healing, Ischemia,
and the Foreign Body Reaction. J. Cell Commun.
Signaling.

[32] Bogdan C. (2015). Nitric Oxide
Synthase in Innate and Adaptive Immunity: An Update. Trends Immunol..

[33] Félétou M., Köhler R., Vanhoutte P. M. (2012). Nitric Oxide: Orchestrator of Endothelium-Dependent
Responses. Ann. Med..

[34] Biswas S. K. (2016). Does the
Interdependence between Oxidative Stress and Inflammation Explain
the Antioxidant Paradox?. Oxid. Med. Cell. Longev..

[35] Torrente L., Prieto-Farigua N., Falzone A., Elkins C. M., Boothman D. A., Haura E. B., DeNicola G. M. (2020). Inhibition of TXNRD or SOD1 Overcomes
NRF2-Mediated Resistance to β-Lapachone. Redox Biol..

[36] Singh B., Bhat H. K. (2012). Superoxide Dismutase 3 Is Induced by Antioxidants,
Inhibits Oxidative DNA Damage and Is Associated with Inhibition of
Estrogen-Induced Breast Cancer. Carcinogenesis.

