# Supplementary material for: Proteomics-Driven Mechanistic Insights into the Anti-Inflammatory Potential of Thinned Apple Polyphenols in a DNBS-Induced Colitis Model in Mice
Source: J Proteome Res. 2026 Jan 26;25(2):684–99. doi: 10.1021/acs.jproteome.5c00653 (PMC12888000; doi:10.1021/acs.jproteome.5c00653)
Supplement: Supplementary file 1 [file pr5c00653_si_001.pdf]

SUPPORTING INFORMATION FOR THE ARTICLE

PROTEOMICS-DRIVEN MECHANISTIC INSIGHTS INTO THE ANTI-INFLAMMATORY POTENTIAL OF THINNED APPLE POLYPHENOLS IN A DNBS-INDUCED COLITIS MODEL IN MICE

Giulio Ferrario[1], Daniela Impellizzeri[2], Giovanna Baron[1], Ramona D'Amico[2], Giulio Fumagalli[1], Tommaso Gnasso[1], Ezio Bombardelli[3], Marina Carini[1], Rosanna di Paola[4], Giancarlo Aldini[1] and Alessandra Altomare[1]*

[1]Department of Pharmaceutical Sciences (DISFARM), Università degli Studi di Milano, Via Mangiagalli 25, 20133 Milano, Italy

[2] Department of Chemical, Biological, Pharmaceutical and Environmental Sciences, University of Messina, Viale F. Stagno D'Alcontres 31, 98166 Messina, Italy

[3] Plantex S.a.s., Galleria Unione 5, 20122 Milano, Italy

[4] Department of Veterinary Sciences, University of Messina, Viale SS Annunziata, 98168 Messina, Italy;.

* Corresponding author: alessandra.altomare@unimi.it; https://orcid.org/0000-0002-9906-6098

Table of contents

Table S1.xlsx Comprehensive list of proteins significantly modulated in at least one of the three pairwise comparisons: DNBS vs CTR, DNBS_TAP vs DNBS, and DNBS_TAP vs CTR.

Figure S1 - Evaluation of the linear correlation of LFQ intensity values between biological and technical replicates ……………………………………………………………………………………………………1

Table S2 - Proteins contributing to the predicted activation of the "Activation of macrophages" function ……………………………………………………………………………………………………....2

Figure S2 - Graphical representation to support the activation hypothesis of IL-6 and its receptor IL-6R, NF-κB and IL-1 in the DNBS vs. SHAM comparison matrix. ………………………………………………3

Table S3 - Proteins contributing to the predicted activation of the upstream regulator IL-6 ……………………………………………………………………………………………………....4

Table S4 - Proteins contributing to the predicted activation of the upstream regulator IL-1 ……………………………………………………………………………………………………8

Table S5 - Proteins contributing to the predicted activation of the upstream regulator IL6R………………...9

Figure S3 - Graphical representation of the 'Quantity of neutrophils' pathway……………………………...11

Table S6 - Proteins contributing to the predicted activation of the "Quantity of neutrophils" function …………………………………………………………………………………………...11

Table S7 - Proteins contributing to the predicted activation of the upstream regulator NFKB……………...13

Figure S4 - List of sub-networks of significantly up-regulated proteins (DNBS vs. SHAM comparison matrix)……………………………………………………………………………………………...14

Table S8 - Proteins contributing to the predicted inhibition of the "Bleeding" function……………………15

Figure S5 - Graphical representation of the 'Inflammation of organ' pathway……………………………...16

Table S9 - Proteins contributing to the predicted activation of the "Inflammation of organ" function……...17

Figure S6 - Graphical representation of the 'Immune response of neutrophils' pathway……………………20

Table S10 - Proteins contributing to the predicted activation of the "Immune response of neutrophils" function……………………………………………………………………………………………20

Table S11 - Proteins contributing to the predicted activation of the Canonical Pathway identified as "Acute Phese Signaling"…………………………………………………………………………………...21

Figure S7 - List of sub-networks of significantly up-regulated proteins (DNBS_TAP vs. DNBS comparison matrix)……………………………………………………………………………………………...24

Figure S1 - Evaluation of the linear correlation of LFQ intensity values between biological and technical replicates by calculating Pearson's linear correlation coefficient.

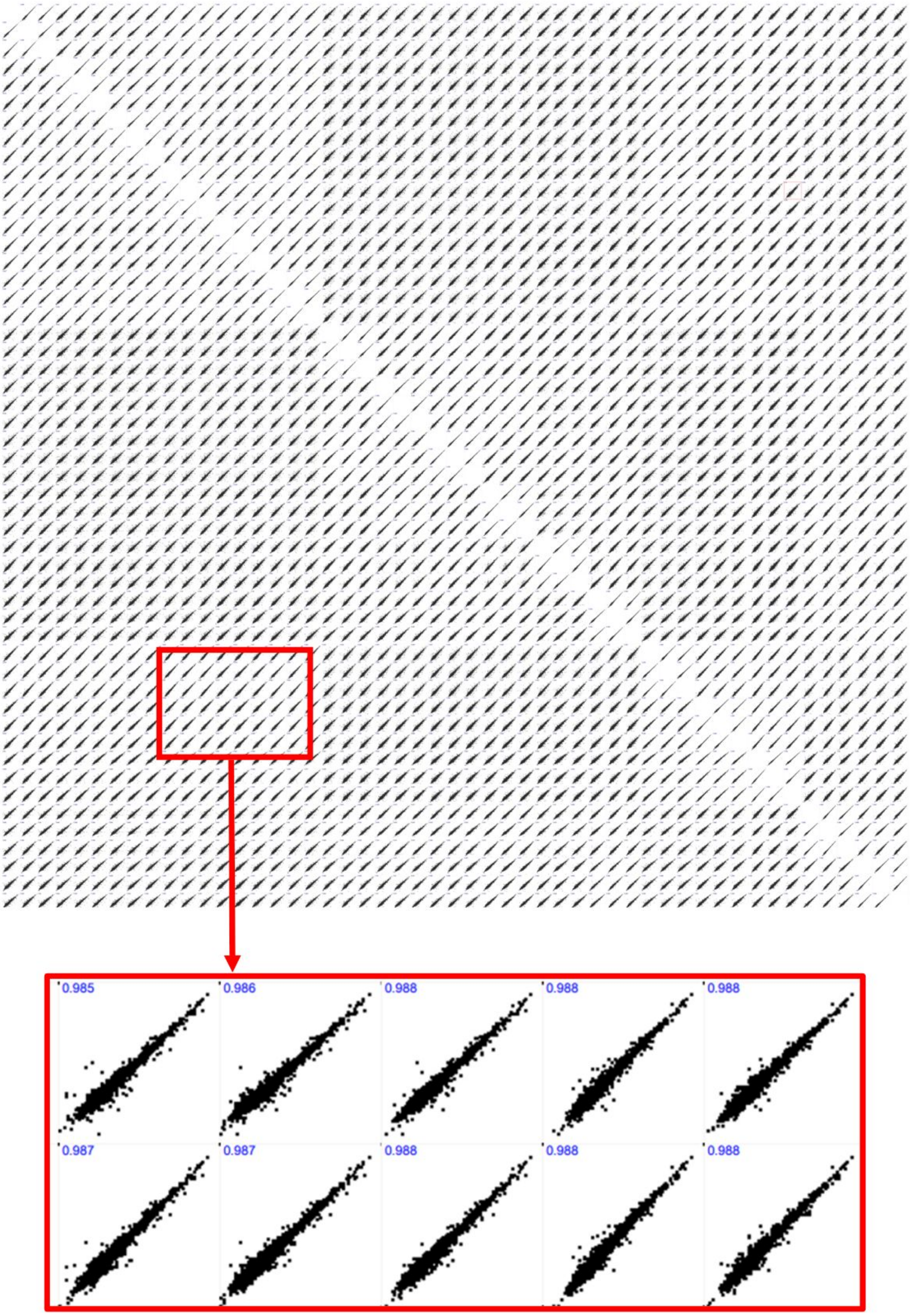

Table S2 - Proteins contributing to the predicted activation of the "Activation of macrophages" function, as identified by Ingenuity Pathway Analysis (IPA) in the Diseases and Functions category. The table lists, for each protein, its expression log ratio, direction of regulation, and related biological findings. Proteins displayed in Figure 5 correspond to those included in this analysis.

© 2000-2025 QIAGEN. All rights reserved.

ID	Genes in dataset	Prediction (based on measurement direction)	Expr Log Ratio	Findings
S100a9	S100A9	Decreased	5,929	Decreases (0)
Vtn	VTN	Increased	5,651	Increases (4)
Thbs1	THBS1	Increased	5,017	Increases (2)
Fgg	FGG	Decreased	4,711	Decreases (1)
Chil3	Chil3/Chil4	Increased	4,34	Increases (1)
Orm1	Orm1 (includes others)	Decreased	3,99	Decreases (0)
Mpo	MPO	Increased	3,833	Increases (1)
Apcs	APCS	Increased	3,778	Increases (1)
Clu	CLU	Affected	3,49	Affects (1)
C4b	C4A/C4B	Increased	2,834	Increases (0)
Kng1	KNG1	Increased	2,719	Increases (0)
Plg	PLG	Affected	2,316	Affects (4)
Fn1	FN1	Increased	2,099	Increases (6)
F2	F2	Increased	1,819	Increases (6)
Ltf	LTF	Increased	1,752	Increases (1)
Serpinf2	SERPINF2	Decreased	1,616	Decreases (1)
C3	C3	Affected	1,572	Affects (2)
Cd14	CD14	Increased	1,438	Increases (2)
Apoh	APOH	Increased	1,326	Increases (2)
Serping1	SERPING1	Decreased	1,32	Decreases (1)
Prdx6	PRDX6	Affected	0,832	Affects (1)
Egfr	EGFR	Increased	0,765	Increases (1)
Itga5	ITGA5	Increased	0,738	Increases (1)
Apoe	APOE	Increased	0,726	Increases (13)
Cd44	CD44	Increased	0,672	Increases (1)
Stk39	STK39	Affected	0,589	Affects (1)
Gc	GC	Increased	0,536	Increases (2)
Hmgb1	Gm21596/Hmgb1	Affected	-0,581	Affects (0)
Adipoq	ADIPOQ	Increased	-0,587	Decreases (2)
Dysf	DYSF	Affected	-0,609	Affects (2)
Ptpn6	PTPN6	Affected	-0,62	Affects (1)
H2-K1	HLA-A	Affected	-0,867	Affects (1)
Clca1	CLCA1	Decreased	-4,176	Increases (1)

Figure S2 - Graphical representation of the subset of proteins that, based on their modulation (up/down-regulation), support the activation hypothesis of IL-6 (A) and its receptor IL-6R (B) in the DNBS vs. SHAM comparison matrix. In panel C of the subset of proteins that support the hypothesis of IL-1 activation, while in panel D of NF-kB pathway.

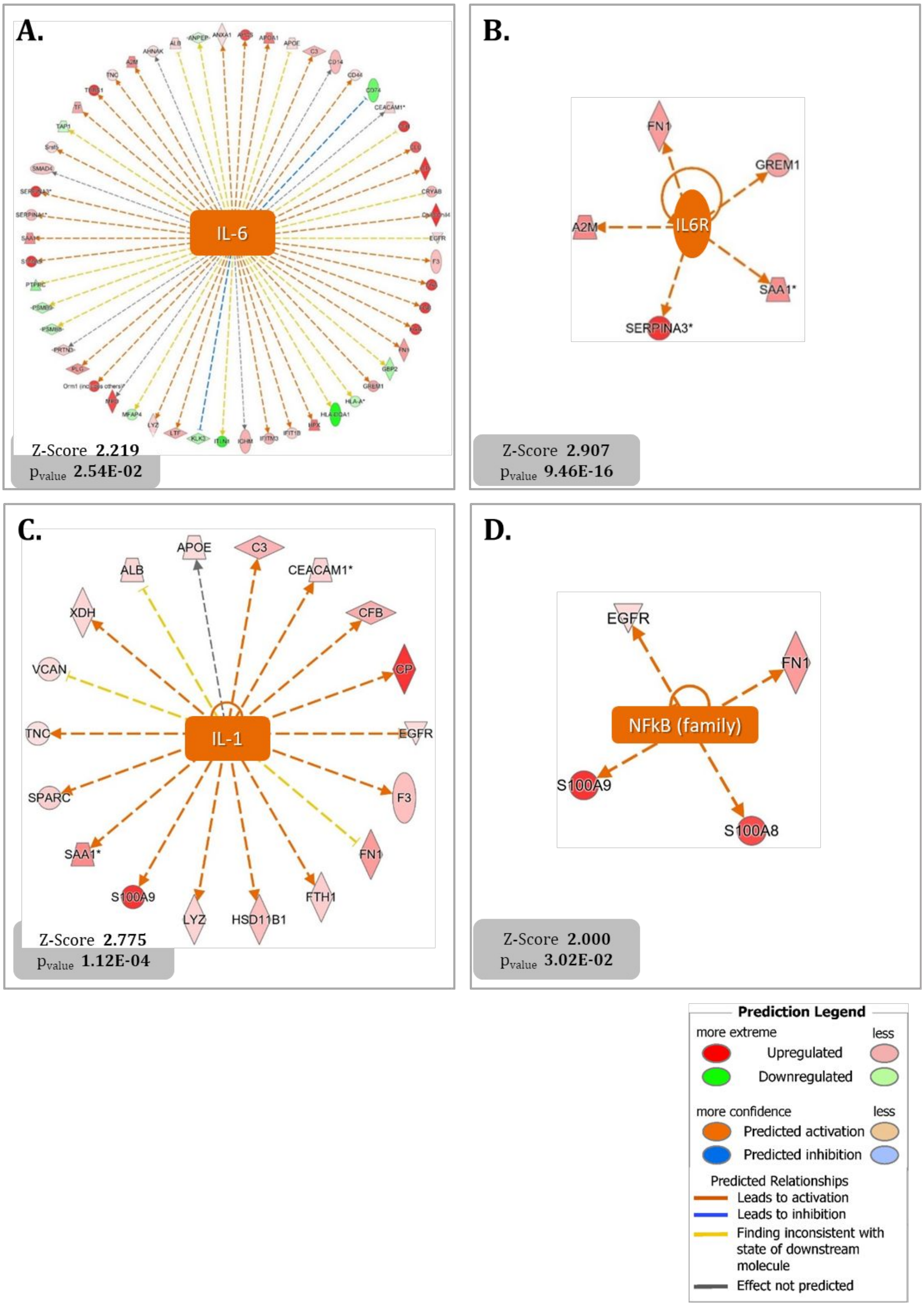

Table S3 - Proteins contributing to the predicted activation of the upstream regulator IL-6, as identified by Ingenuity Pathway Analysis (IPA). The table reports, for each protein, gene symbol, expression value (log ratio and p-value), subcellular localization, and functional family. Proteins shown correspond to those supporting the activation of IL-6 reported in Figure S2_A.

© 2000-2025 QIAGEN. All rights reserved.

Symbol	Synonym(s)	Entrez Gene Name	Gene Symbol - human (HUGO / HGNC / Entrez Gene)/Gene Symbol - mouse (Entrez Gene)	Expr p-value	Expr Log Ratio	*Location*	*Family*
A2M			A2m	7,87877E-19	2,503101179	*Extracellular Space*	*transporter*
AHNAK	1110004P15RIK, 2310047C17RIK, AHNAK 1, AHNAK nucleoprotein, AHNAKRS, AHNK, DA, desmoyokin, DY6, FLJ33834, Hypothetical PDZ Domain, LOC686579, MGC5395, PM227, RGD1308572, UV118	AHNAK nucleoprotein	Ahnak	3,59303E-12	0,550654708	*Nucleus*	*other*
ALB	Alb-1, Albumin, Albumin 1, albumin isomer 1, albumin isomer 2, albumin isomer 3, Albza, BCL002, FDAHT, HSA, PRO0883, PRO0903, PRO1341, PRO2044, SERUM ALBUMIN, serum albumin chain A, Serum albumin precursor	albumin	Alb	3,45982E-07	0,846567027	*Extracellular Space*	*transporter*
ANPEP	AAP, Ala-(Leu-Gly)-Aminopeptidase, Alanine Aminopeptidase, alanyl aminopeptidase, membrane, AMINOPEPTIDASE M, Aminopeptidase n, AP-M, AP-N, CD13, GP150, hAPN, Kidney Aminopeptidase M, KZP, LAP1, P150, PEPN, rAPN	alanyl aminopeptidase, membrane	Anpep	5,76722E-08	-0,503755103	*Plasma Membrane*	*peptidase*
ANXA1	Annexin-1, annexin A1, ANX1, C430014K04RIK, Lc1, Lipocortin, Lipocortin-1, LPC1, p35	annexin A1	Anxa1	2,77939E-07	0,548888185	*Plasma Membrane*	*other*
APCS	amyloid P component, serum, HEL-S-92n, PTX2, SAP, Serum Amyloid P, Serum amyloid p-component	amyloid P component, serum	Apcs	1,3743E-10	3,778403409	*Extracellular Space*	*other*
APOA1	Alp-1, AMYLD3, Apo1a, APO A, apo(a), APOA-I, APOLIPOPROTEIN A1, APOLIPOPROTEIN A-I, APOLIPOPROTEIN A-I G1, Brp-14, CSL112, HPALP2, LP(A-I), Ltw-1, Lvtw-1, Sep-1, Sep-2	apolipoprotein A1	Apoa1	2,8233E-09	2,811790254	*Extracellular Space*	*transporter*
APOE	AD2, APOEA, Apolipoprotein E, LDLCQ5, LPG	apolipoprotein E	Apoe	2,93306E-07	0,725990295	*Extracellular Space*	*transporter*
C3	ACYLATION STIMULATING, AHUS5, ARMD9, ASP, circC3, circRNA-C3, Complement c3, complement component 3, CPAMD1, HEL-S-62p, HSE-MSF, Plp	complement C3	C3	2,72588E-15	1,572466744	*Extracellular Space*	*peptidase*
CD14	CD14 ANTIGEN, CD14 molecule, lipopolysaccharide (LPS) receptor	CD14 molecule	Cd14	4,51515E-10	1,437866783	*Plasma Membrane*	*transmembrane receptor*
CD44	216062 AT, CD44A, CD44 Antigen, CD44 (containing exon 5), Cd44i, CD44 molecule, CD44 molecule (IN blood group), CD44 STANDARD FROM, CDW44, CSPG8, ECM-III, ECMR-III, Epican, H-CAM, HCELL, HERMES, Hermes-1, Hermes antigen, HUTCH-1, HUTCH-I, IN, LHR, Ly-24, MC56, MDU2, MDU3, METAA, MIC4, NKT.44, Pgp-1, RHAMM	CD44 molecule (IN blood group)	Cd44	4,36682E-08	0,672130881	*Plasma Membrane*	*other*
CD74	CD74 ANTIGEN, CD74 antigen (invariant polypeptide of major histocompatibility complex, class II antigen-associated), CD74 molecule, CLIP, DHLAG, HLADG, Hla-dr γ, Ia-associated invariant chain, Ia-GAMMA, Ia-γ, II, INVARIANT CHAIN, INVG34, MHC Class2 Associated Invariant Chain, MHC Class I IIDG, MHCII (I-A), MIF receptor, Ox6, p33	CD74 molecule	Cd74	0,00172651	-1,83891858	*Plasma Membrane*	*transmembrane receptor*

CEACAM1			Ceacam2	0,013986045	0,932593664	*Plasma Membrane*	*transporter*
CFH			Cfh	3,1892E-18	4,773623806	*Extracellular Space*	*other*
Chil3/Chil4	Chi3l3, Chi3l4, Chil3, Chil4, Chitinase 3-like 3, Chitinase 3-like 4, chitinase-like 3, chitinase-like 4, ECF-L, rYM1olf, Ym1, Ym2	chitinase-like 3	Chil3	2,0166E-06	4,33986098	*Cytoplasm*	*enzyme*
CLU	AAG4, APO-J, CLI, CLUSTERIN, COMPLEMENT CYTOLYSIS INHIBITOR, D14Ucla3, DAG, Gp80, KUB1, NA1/NA2, RATTRPM2B, SGP-2, SP-40, Sugp-2, Sulfated glycoprotein 2, sulphated glycoprotein 2, TRPM-2, TRPM2B, Trpmb	clusterin	Clu	3,76044E-14	3,489597012	*Cytoplasm*	*other*
CP	AB073614, CERP, ceruloplasmin, CP-2, D3Ertd555e, Ferroxidase, FOX	ceruloplasmin	Cp	1,80159E-25	4,352745734	*Extracellular Space*	*enzyme*
CRYAB	AACRYA, ABC, alpha B CRYSTALLIN, Alpha crystallin b chain, CMD1II, CRYA2, CRYSTALLIN alpha 2, Crystallin alpha b, crystallin, alpha B, CRYSTALLIN α 2, Crystallin α B, Crystallin, α B, CTPP2, CTRCT16, Heat-shock 20 kDa like, HEL-S-101, HSPB5, MFM2, P23, α B CRYSTALLIN, α crystallin b chain, α β crystallin	crystallin alpha B	Cryab	1,16062E-19	1,641595798	*Nucleus*	*other*
EGFR	9030024J15RIK, C-ERBB, EGFR1, EGF receptor, EGFR vIII, EGF/TGF-alpha RTK, EGF/TGF-α RTK, EGF-TK, epidermal growth factor receptor, ERBB, ERBB1, Errb1, ERRP, HER1, HER1 (EGFR), MENA, NISBD2, NNCIS, PIG61, wa-2, Wa5	epidermal growth factor receptor	Egfr	1,81634E-05	0,764930998	*Plasma Membrane*	*kinase*
F3	AA409063, CD142, Cf-3, coagulation factor III, coagulation factor III, tissue factor, TF	coagulation factor III, tissue factor	F3	3,33639E-07	1,324693898	*Plasma Membrane*	*transmembrane receptor*
FGA	Aalpha-fibrinogen, Ac1873, AI303526, alpha-fibrinogen, AMYLD2, FAC, Fba5e, Fib, Fib2, Fibrinogen a, Fibrinogen alpha, fibrinogen alpha chain, Fibrinogen A α, Fibrinogen α, fibrinogen α chain	fibrinogen alpha chain	Fga	5,25598E-26	4,877095858	*Extracellular Space*	*other*
FGB	2510049G14Rik, Ab1-181, Ab1-216, Ac1-581, Beta fibrinogen, FBC, Fg beta chain, Fg β chain, FIBRINOGEN beta CHAIN, Fibrinogen b β, FIBRINOGEN β CHAIN, Fibronogen B β, HEL-S-78p, β-fibrinogen	fibrinogen beta chain	Fgb	6,03649E-23	5,093109745	*Extracellular Space*	*other*
FGG	3010002H13Rik, Fg gamma, Fg gamma chain, FGG isoform 1, Fg γ, Fg γ chain, fibrinogen gamma, fibrinogen gamma chain, Fibrinogen γ, Fibrinogen γ chain, PRO2061	fibrinogen gamma chain	Fgg	1,31469E-25	4,7109343	*Extracellular Space*	*other*
FN1	cFn, CIG, E330027I09, ED-B, FIBNEC, Fibronectin, FIBRONECTIN 1, Fibronectin3 M1, Fibronectin i, FINC, FN, FN1 isoform 1, FNZ, GFND, GFND2, LETS, MSF, SMDCF	fibronectin 1	Fn1	5,57517E-05	2,098632336	*Extracellular Space*	*other*
GBP2			Gbp2	1,42638E-06	-1,098874961	*Cytoplasm*	*enzyme*
GREM1	C15DUPq, CKTSF1B1, CRAC1, CRCS4, DAND2, DRM, DUP15q, Grem, GREMLIN, GREMLIN 1, gremlin 1, DAN family BMP antagonist, HMPS, HMPS1, IHG-2, Id, MPSH, PIG2	gremlin 1, DAN family BMP antagonist	Grem1	7,45274E-14	1,995183249	*Extracellular Space*	*other*

HLA-A	0610037M15Rik, A-28, Aw-24, Aw-33, Aw-34, Aw-66, Aw-68, Aw-69, Aw-74, Aw-80, BE136769, EG667977, ENSMUSG00000121510, Gm10499, Gm11132, Gm8909, gs14-2, H2-B1, H2-Bl, H2-Bl-like, H-2D, H2-D1, H-2DD, H2-Gs10, H2-gs17, H-2K, H2-K1, H-2K(d), H-2Kd, H2-Q1, H2-Q10, H2-Q2, H2-Q4, H2-Q6, H2-Q7, H2-T13, H2-T26, H2-T5, H2-T7, H2-Tw5l, histocompatibility 2, blastocyst, histocompatibility 2, D region locus 1, histocompatibility 2, K1, K region, histocompatibility 2, Q region locus 1, histocompatibility 2, Q region locus 10, histocompatibility 2, Q region locus 2, histocompatibility 2, Q region locus 4, histocompatibility 2, Q region locus 6, histocompatibility 2, Q region locus 7, histocompatibility 2, T region locus 26, HLA-C, HLA class I histocompatibility antigen A-23 alpha chain, HLA class I histocompatibility antigen A-23 α chain, Kb, Kd h chain, K-f, LOC100045864, LOC100133382, LOC100364500, LOC100365191, LOC100507703, LOC100862431, LOC100862447, LOC102554995, LOC102557454, LOC103690108, LOC108167390, LOC108348299, LOC108349031, LOC108349033, LOC56628, LOC636948, LOC684280, LOC687138, major histocompatibility complex, class I, A, MHC 1alpha, MHC-A, Mhc class I q2-k antigen, MHC I, Mhc-i-kd, MumuTL, Ped, predicted gene 10499, Q1, Q10, Q1b, Q1d, Q1k, Q9, Qa-1, Qa10, Qa-2, Qa-4, Qa-6, Qa-7, Qat-4, Qb-1, Qed-1, RT1-A, RT1-A1, RT1.A1(N), RT1-A1n, RT1-A2, RT1.A2(N), RT1-A2n, RT1-C/E1, RT1-CE1, RT1-CE10, RT1-CE12, RT1-CE13, RT1-CE15, RT1-CE16, RT1-C/E2, RT1-CE2, RT1-C/E3, RT1-CE3, RT1-C/E4, RT1-CE4, RT1-C/E5, RT1-CE5, RT1-C/E7, RT1-CE7, RT1.Cg, RT1 class Ia, locus A1, RT1 class Ia, locus A2, RT1 class I, locus1, RT1 class I, locus CE10, RT1 class I, locus CE13, RT1 class I, locus CE15, RT1 class I, locus CE16, RT1 class I, locus CE2, RT1 class I, locus CE3, RT1 class I, locus CE4, RT1 class I, locus CE5, RT1 class I, locus CE7, RT1-E2, rt1-El, RT1-Uav1, RT1-Uc, RT1-Ulv1	major histocompatibility complex, class I, A	H2-K1	0,000110645	-0,86671132	*Plasma Membrane*	*other*
HLA-DQA1			H2-Aa	1,95139E-07	-2,815380838	*Plasma Membrane*	*transmembrane receptor*
HPX	haemo, haemopexin, HEMO, hemopexin, HPXN, HX	hemopexin	Hpx	1,22444E-23	2,989355469	*Extracellular Space*	*transporter*
IFIT1B			Ifit1	0,008219082	0,988780181	*Cytoplasm*	*other*
IFITM3			Ifitm3	4,37604E-12	1,582865122	*Plasma Membrane*	*other*
IGHM	AGM1, Ak007163, cyto-Ig mu, FLJ00385, IG gamma-1 chain c region, IGH-6, IG HEAVY CONSTANT MU, IgM, IgM mu, Ig mu, Ig mu chain c region, IG γ-1 chain c region, Immunoglobulin heavy chain 6, immunoglobulin heavy constant mu, IPI00022910, MU, Mu chain, muH, muHC, Mu heavy chain, Mu MT, Mu S, VH, VH186.2	immunoglobulin heavy constant mu	Ighm	1,59535E-18	1,5816384	*Plasma Membrane*	*transmembrane receptor*
IL6	BSF-2, CDF, FDGI, HGF, HSF, IFNB2, IFN-beta-2, IFN beta 2A, IFN-β-2, IFN β 2A, ILg6, interleukin-6	interleukin 6				*Extracellular Space*	*cytokine*
ITLN1	FLJ20022, hIntL, HL-1, intelectin 1, intelectin 1 (galactofuranose binding), INTL, ITLN, Itln2, Itln3, Itln5, Itlna, Lactoferrin receptor, LFR, LOC100363780, Omentin, Omentin-1, OMNT1	intelectin 1	Itln1	1,24948E-10	-3,441832648	*Plasma Membrane*	*other*
KLK3			Klk1	0,000116396	-1,06754161	*Extracellular Space*	*peptidase*
LTF	Csp82, GIG12, HEL110, HLF2, Lactoferrin, lactotransferrin, LF, LOC102724305, MMS10R, Ms10r, TRFL	lactotransferrin	Ltf	0,009618714	1,752218628	*Extracellular Space*	*other*

LYZ			Lyz2	0,002606217	0,911704614	*Extracellular Space*	*enzyme*
MFAP4	1110007F23RIK, LOC100911714, LOC102553715, Magp-36, microfibril associated protein 4, microfibrillar-associated protein 4	microfibril associated protein 4	Mfap4	1,17892E-09	-0,925695525	*Extracellular Space*	*other*
MPO	mKIAA4033, myeloperoxidase	myeloperoxidase	Mpo	2,22778E-05	3,832584429	*Cytoplasm*	*enzyme*
Orm1 (includes others)			Orm1	3,41015E-07	3,990268135	*Extracellular Space*	*other*
PLG	Ab1-346, GLU-PG, HAE4, LPA, Pg, PG2, plasminogen, Scdp	plasminogen	Plg	5,45097E-16	2,315633816	*Extracellular Space*	*peptidase*
PRTN3			Prtn3	0,021548715	1,087139448	*Extracellular Space*	*peptidase*
PSMB8	20s proteosome subunit, ALDD, Beta 5i, beta 5I IMMUNOPROTEASOME subunit, D6S216, D6S216E, JMP, large multifunctional protease-7, LMP7, Lmp8, LOC103690099, NKJO, PRAAS1, proteasome 20S subunit beta 8, proteasome 20S subunit β 8, proteasome (prosome, macropain) subunit, beta type 8 (large multifunctional peptidase 7), proteasome (prosome, macropain) subunit, β type 8 (large multifunctional peptidase 7), proteasome subunit Y, PSMB5i, Rc1, RING10, β 5i, β 5I IMMUNOPROTEASOME subunit	proteasome 20S subunit beta 8	Psmb8	4,84165E-05	-0,91111092	*Cytoplasm*	*peptidase*
PSMB9	Beta 1i, beta 1I IMMUNOPROTEASOME subunit, large multifunctional protease 2, LMP2, PRAAS3, PRAAS6, proteasome 20S subunit beta 9, proteasome 20S subunit β 9, proteasome (prosome, macropain) subunit, beta type 9 (large multifunctional peptidase 2), proteasome (prosome, macropain) subunit, β type 9 (large multifunctional peptidase 2), proteasome subunit X, Proteasome subunit, β type, 9, Psamb9, PSMB6i, RING12, β 1i, β 1I IMMUNOPROTEASOME subunit	proteasome 20S subunit beta 9	Psmb9	3,30931E-06	-0,943181017	*Cytoplasm*	*peptidase*
PTPRC	B220, CD45, CD45R, GP180, IMD105, L-CA, loc, LY5, Lyt-4, Ox1, protein tyrosine phosphatase receptor type C, protein tyrosine phosphatase, receptor type, C, RT7, T200	protein tyrosine phosphatase receptor type C	Ptprc	1,57763E-07	-1,388676728	*Plasma Membrane*	*phosphatase*
S100A9			S100a9	8,93565E-09	5,928692945	*Cytoplasm*	*other*
SAA1			Saa1	1,73834E-05	2,427020963	*Extracellular Space*	*transporter*
SERPINA1			Serpina1a	4,07068E-13	1,458146265	*Extracellular Space*	*other*
SERPINA3			Serpina3n	1,13995E-20	5,567598597	*Extracellular Space*	*other*
SMAD4	AW743858, D18Wsu70e, DPC4, JIP, MADH4, MYHRS, SMAD family member 4, Smaug1	SMAD family member 4	Smad4	8,92504E-08	1,197676204	*Nucleus*	*transcription regulator*
Srsf5			Srsf5	1,38066E-09	0,707017181	*Nucleus*	*other*
TAP1	ABC17, ABCB2, APT1, Cim, D6S114E, Ham-1, MHC1D1, MTP1, PSF-1, RING4, TAP, TAP1*0102N, TAP1N, Tap2, TRANSPORTER 1 ATP-binding CASSETTE SUBFAMILY B, transporter 1, ATP-binding cassette, sub-family B (MDR/TAP), transporter 1, ATP binding cassette subfamily B member, TRANSPORTER 1 (MDR/TAP), Y3	transporter 1, ATP binding cassette subfamily B member	Tap1	0,014231724	-0,680106864	*Cytoplasm*	*transporter*
TF	Apo-Tf, Apo-transferrin, Cd176, HEL-S-71p, Holo-Tf, Holo-transferrin, HP, hpx, Liver regeneration related protein lrrg03, Liver Reneration-related, PRO1557, PRO2086, Serotransferrin, Serotransferrin precursor, Tfn, TFQTL1, TRANSFERRIN, Trf	transferrin	Trf	9,26586E-13	1,799203195	*Extracellular Space*	*transporter*
THBS1	tbsp1, THBS, thrombospondin 1, THSP, TS-1, TSP, TSP-1	thrombospondin 1	Thbs1	9,79954E-12	5,016660372	*Extracellular Space*	*other*

TNC	150-225, C130033P17Rik, cytotactin, DFNA56, GMEM, GP, HXB, JI, Ten, tenascin-C, TN	tenascin C	Tnc	0,000299392	0,612409952	*Extracellular Space*	*other*

Table S4 - Proteins contributing to the predicted activation of the upstream regulator IL-1, as identified by Ingenuity Pathway Analysis (IPA). The table reports, for each protein, gene symbol, expression value (log ratio and p-value), subcellular localization, and functional family. Proteins shown correspond to those supporting the activation of IL-1 reported in Figure S2_C.

© 2000-2025 QIAGEN. All rights reserved.

Symbol	Synonym(s)	Entrez Gene Name	Gene Symbol - human (HUGO / HGNC / Entrez Gene)/Gene Symbol - mouse (Entrez Gene)	Expr p-value	Expr Log Ratio	Location	Family
ALB	Alb-1, Albumin, Albumin 1, albumin isomer 1, albumin isomer 2, albumin isomer 3, Albza, BCL002, FDAHT, HSA, PRO0883, PRO0903, PRO1341, PRO2044, SERUM ALBUMIN, serum albumin chain A, Serum albumin precursor	albumin	Alb	3,45982E-07	0,846567027	*Extracellular Space*	*transporter*
APOE	AD2, APOEA, Apolipoprotein E, LDLCQ5, LPG	apolipoprotein E	Apoe	2,93306E-07	0,725990295	*Extracellular Space*	*transporter*
C3	ACYLATION STIMULATING, AHUS5, ARMD9, ASP, circC3, circRNA-C3, Complement c3, complement component 3, CPAMD1, HEL-S-62p, HSE-MSF, Plp	complement C3	C3	2,72588E-15	1,572466744	*Extracellular Space*	*peptidase*
CEACAM1			Ceacam2	0,013986045	0,932593664	*Plasma Membrane*	*transporter*
CFB	AHUS4, AI255840, ARMD14, BF, BFD, C2, CFAB, CFBD, Complement Factor B, Da1-24, Factor B, FB, FBI12, GBG, H2-Bf, PBF2	complement factor B	Cfb	1,16618E-14	1,720757696	*Extracellular Space*	*peptidase*
CP	AB073614, CERP, ceruloplasmin, CP-2, D3Ertd555e, Ferroxidase, FOX	ceruloplasmin	Cp	1,80159E-25	4,352745734	*Extracellular Space*	*enzyme*
EGFR	9030024J15RIK, C-ERBB, EGFR1, EGF receptor, EGFR vIII, EGF/TGF-alpha RTK, EGF/TGF-α RTK, EGF-TK, epidermal growth factor receptor, ERBB, ERBB1, Errb1, ERRP, HER1, HER1 (EGFR), MENA, NISBD2, NNCIS, PIG61, wa-2, Wa5	epidermal growth factor receptor	Egfr	1,81634E-05	0,764930998	*Plasma Membrane*	*kinase*
F3	AA409063, CD142, Cf-3, coagulation factor III, coagulation factor III, tissue factor, TF	coagulation factor III, tissue factor	F3	3,33639E-07	1,324693898	*Plasma Membrane*	*transmembrane receptor*
FN1	cFn, CIG, E330027I09, ED-B, FIBNEC, Fibronectin, FIBRONECTIN 1, Fibronectin3 M1, Fibronectin i, FINC, FN, FN1 isoform 1, FNZ, GFND, GFND2, LETS, MSF, SMDCF	fibronectin 1	Fn1	5,57517E-05	2,098632336	*Extracellular Space*	*other*
FTH1	APOFERRITIN H CHAIN, Ferritin heavy chain, ferritin heavy chain 1, ferritin heavy polypeptide 1, Ferritin subunit H, FHC, Frih, FTH, FTHL6, HFE5, H-ferritin, HFt, MFH, NBIA9, PIG15, PLIF	ferritin heavy chain 1	Fth1	3,09792E-10	1,038545757	*Cytoplasm*	*enzyme*
HSD11B1	11 beta HSD, 11-beta-HSD1, 11-DH, 11 β HSD, 11-β HSD-1, CORTRD2, HDL, HSD11, HSD11B, HSD11L, Hsdrla, hydroxysteroid 11-beta dehydrogenase 1, hydroxysteroid 11-β dehydrogenase 1, LRRGT00065, SDR26C1	hydroxysteroid 11-beta dehydrogenase 1	Hsd11b1	3,0246E-05	1,330398178	*Cytoplasm*	*enzyme*
IL1 (family)	Interleukin-1					*Extracellular Space*	*group*
LYZ			Lyz2	0,002606217	0,911704614	*Extracellular Space*	*enzyme*
S100A9			S100a9	8,93565E-09	5,928692945	*Cytoplasm*	*other*
SAA1			Saa1	1,73834E-05	2,427020963	*Extracellular Space*	*transporter*

SPARC	Basement membrane protein-40, BM-40, OI17, ON, ONT, secreted acidic cysteine rich glycoprotein, secreted protein acidic and cysteine rich	secreted protein acidic and cysteine rich	Sparc	1,15219E-08	1,043014018	*Extracellular Space*	*other*
TNC	150-225, C130033P17Rik, cytotactin, DFNA56, GMEM, GP, HXB, JI, Ten, tenascin-C, TN	tenascin C	Tnc	0,000299392	0,612409952	*Extracellular Space*	*other*
VCAN	5430420N07RIK, 9430051N09, CHONDROITIN SULFATE PROTEOGLYCAN, Chondroitin sulfate proteoglycan 2, CHONDROITIN sulphATE PROTEOGLYCAN, Chondroitin sulphate proteoglycan 2, CSPG2, DPEAAE, ERVR, GHAP, hdf, NG2, PG-M, PG-M(V0), PG-M(V1), Versican, Versican V1, Versican V2, WGN, WGN1	versican	Vcan	1,54013E-07	0,711909972	*Extracellular Space*	*other*
XDH	XAN1, Xanthine Dehydrogenase, Xanthine Oxidase, XO, XOR, Xox-1	xanthine dehydrogenase	Xdh	4,48014E-16	0,843543434	*Cytoplasm*	*enzyme*

Table S5 - Proteins contributing to the predicted activation of the upstream regulator IL6R, as identified by Ingenuity Pathway Analysis (IPA). The table reports, for each protein, gene symbol, expression value (log ratio and p-value), subcellular localization, and functional family. Proteins shown correspond to those supporting the activation of IL6R reported in Figure S2_B.

© 2000-2025 QIAGEN. All rights reserved.

Symbol	Synonym(s)	Entrez Gene Name	Gene Symbol - human (HUGO / HGNC / Entrez Gene)/Gene Symbol - mouse (Entrez Gene)	Expr p-value	Expr Log Ratio	Location	Family
A2M			A2m	7,87877E-19	2,503101179	*Extracellular Space*	*transporter*
FN1	cFn, CIG, E330027I09, ED-B, FIBNEC, Fibronectin, FIBRONECTIN 1, Fibronectin3 M1, Fibronectin i, FINC, FN, FN1 isoform 1, FNZ, GFND, GFND2, LETS, MSF, SMDCF	fibronectin 1	Fn1	5,57517E-05	2,098632336	*Extracellular Space*	*other*
GREM1	C15DUPq, CKTSF1B1, CRAC1, CRCS4, DAND2, DRM, DUP15q, Grem, GREMLIN, GREMLIN 1, gremlin 1, DAN family BMP antagonist, HMPS1, HMPS, IHG-2, Id, MPSH, PIG2	gremlin 1, DAN family BMP antagonist	Grem1	7,45274E-14	1,995183249	*Extracellular Space*	*other*
IL6R	CD126, Gp80, HIES5, IL-1Ra, IL6Q, IL6QTL, IL-6R-1, IL-6RA, IL-6R-alpha, Il6 receptor, IL6RQ, IL-6R-α, interleukin 6 receptor, interleukin 6 receptor, alpha, INTERLEUKIN-6 receptors, interleukin 6 receptor, α, Interleukin 6 receptor α chain	interleukin 6 receptor				*Plasma Membrane*	*transmembrane receptor*
SAA1			Saa1	1,73834E-05	2,427020963	*Extracellular Space*	*transporter*
SERPINA3			Serpina3n	1,13995E-20	5,567598597	*Extracellular Space*	*other*

Figure S3 - Graphical representation of the 'Quantity of neutrophils' pathway for which IPA predicts significant activation with a Z- SCORE value of 2.476. The set of genes up (red) and down (green) regulated to support the generated hypothesis are shown at the ends of the wheel graph.

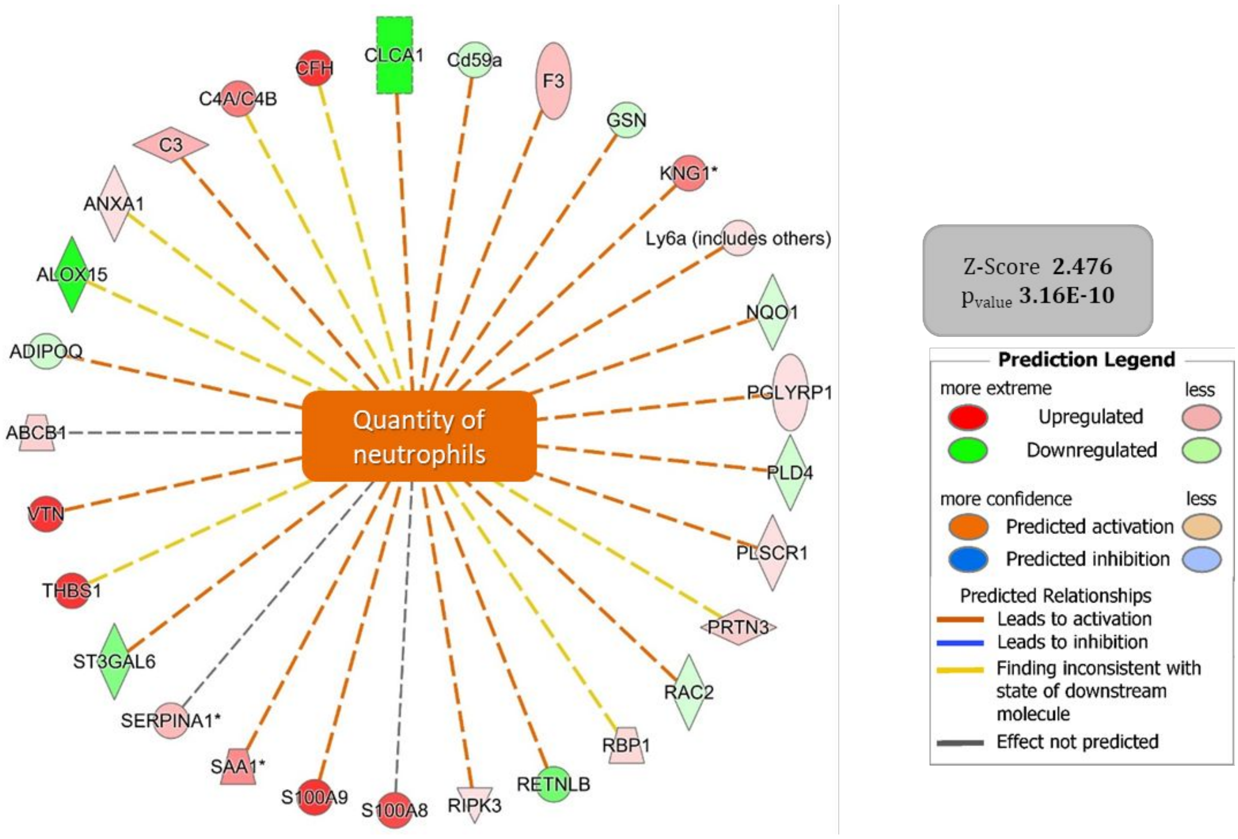

Table S6 - Proteins contributing to the predicted activation of the "Quantity of neutrophils" function, as identified by Ingenuity Pathway Analysis (IPA) in the Diseases and Functions category. The table lists, for each protein, its expression log ratio, direction of regulation, and related biological findings. Proteins displayed in Figure S3 correspond to those included in this analysis.

© 2000-2025 QIAGEN. All rights reserved.

ID	Genes in dataset	Prediction (based on measurement direction)	Expr Log Ratio	Findings
S100a9	S100A9	Increased	5,929	Increases (0)
Vtn	VTN	Increased	5,651	Increases (2)
Thbs1	THBS1	Decreased	5,017	Decreases (1)
Cfh	CFH	Decreased	4,774	Decreases (0)
S100a8	S100A8	Affected	3,818	Affects (2)
C4b	C4A/C4B	Decreased	2,834	Decreases (0)
Kng1	KNG1	Increased	2,719	Increases (0)
Saa1	SAA1	Increased	2,427	Increases (0)
C3	C3	Increased	1,572	Increases (3)
Serpina1a	SERPINA1	Affected	1,458	Affects (0)
F3	F3	Increased	1,325	Increases (1)

Prtn3	PRTN3	Decreased	1,087	Decreases (0)
Abcb1a	ABCB1	Affected	0,969	Affects (0)
Rbp1	RBP1	Decreased	0,826	Decreases (1)
Ripk3	RIPK3	Increased	0,649	Increases (2)
Plscr1	PLSCR1	Increased	0,612	Increases (0)
Pglyrp1	PGLYRP1	Increased	0,562	Increases (2)
Anxa1	ANXA1	Decreased	0,549	Decreases (1)
Ly6a	Ly6a (includes others)	Increased	0,511	Increases (1)
Nqo1	NQO1	Increased	-0,502	Decreases (4)
Rac2	RAC2	Increased	-0,512	Decreases (4)
Pld4	PLD4	Increased	-0,558	Decreases (1)
Adipoq	ADIPOQ	Increased	-0,587	Decreases (2)
Gsn	GSN	Increased	-0,647	Decreases (1)
Cd59a	Cd59a	Increased	-0,693	Decreases (1)
St3gal6	ST3GAL6	Increased	-1,537	Decreases (1)
Retnlb	RETNLB	Increased	-1,778	Decreases (0)
Alox15	ALOX15	Decreased	-3,367	Increases (0)
Clca1	CLCA1	Increased	-4,176	Decreases (1)

Table S7 - Proteins contributing to the predicted activation of the upstream regulator NFKB, as identified by Ingenuity Pathway Analysis (IPA). The table reports, for each protein, gene symbol, expression value (log ratio and p-value), subcellular localization, and functional family. Proteins shown correspond to those supporting the activation of NFKB reported in Figure S2_D.

© 2000-2025 QIAGEN. All rights reserved.

Symbol	Synonym(s)	Entrez Gene Name	Gene Symbol - human (HUGO / HGNC / Entrez Gene)/Gene Symbol - mouse (Entrez Gene)	Expr p-value	Expr Log Ratio	Location	Family
EGFR	9030024J15RIK, C-ERBB, EGFR1, EGF receptor, EGFR vIII, EGF/TGF-alpha RTK, EGF/TGF-α RTK, EGF-TK, epidermal growth factor receptor, ERBB, ERBB1, Errb1, ERRP, HER1, HER1 (EGFR), MENA, NISBD2, NNCIS, PIG61, wa-2, Wa5	epidermal growth factor receptor	Egfr	1,81634E-05	0,764930998	*Plasma Membrane*	*kinase*
FN1	cFn, CIG, E330027I09, ED-B, FIBNEC, Fibronectin, FIBRONECTIN 1, Fibronectin3 M1, Fibronectin i, FINC, FN, FN1 isoform 1, FNZ, GFND, GFND2, LETS, MSF, SMDCF	fibronectin 1	Fn1	5,57517E-05	2,098632336	*Extracellular Space*	*other*
NFkB (family)	NF Kappa B, NF-kappaB p50/p52, NF-κB					*Nucleus*	*group*
S100A8	60B8AG, B8Ag, CAGA, calgranulin A, CALPROTECTIN, CFAG, CGLA, CP-10, CYSTIC FIBROSIS ANTIGEN, L1Ag, MA387, MIF, MIGRATION INHIBITORY FACTOR RELATED protein 8, MRP8, NIF, P8, PM-2K, S100 calcium binding protein A8, S100 calcium binding protein A8 (calgranulin A)	S100 calcium binding protein A8	S100a8	2,99376E-09	3,818053913	*Cytoplasm*	*other*
S100A9			S100a9	8,93565E-09	5,928692945	*Cytoplasm*	*other*

Figure S4 - List of sub-networks of significantly up-regulated proteins (DNBS vs. SHAM comparison matrix), united by their involvement in specific biological processes; below the list of protein involved is the color code that associates each gene product with the GO (gene ontology annotation) used for functional enrichment.

Subnetwork A

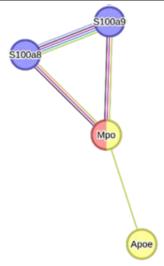

Protein name	Gene name	-Log pvalue	Ratio DNBS vs SHAM
Apolipoprotein E	Apoe	6,532	0.725
Myeloperoxidase	Mpo	4,652	3,832
Protein S100-A8	S100a8	8,524	3,819
Protein S100-A9	S100a9	8,049	5,929

Color code:
Response to oxidative stress
Hydrogen peroxide catabolic process
Toll-like receptor 4 binding

Subnetwork B

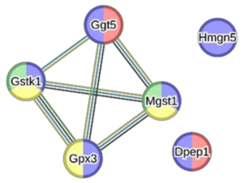

Protein name	Gene name	-Log pvalue	Ratio DNBS vs SHAM
Gamma-glutamyl-transferase 5	Ggt5	9,999	0.540
Glutathione S-transferase kappa 1	Gstk1	7,993	0.644
Glutathione peroxidase 3	Gpx3	7,285	0.539
Microsomal glutathione S-transferase 1	Mgst1	5,326	0.570
High mobility group nucleosome-binding domain-containing protein 5	Hmgn5	2,834	0.621
Dipeptidase 1	Dpep1	3,591	0.544

Color code:
Glutathione catabolic process
Glutathione metabolic process
Glutathione peroxidase activity
Glutathione transferase activity

Subnetwork C

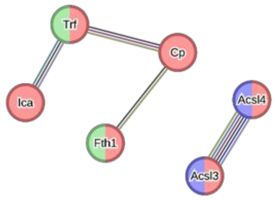

Protein name	Gene name	-Log pvalue	Ratio DNBS vs SHAM
Serotransferrin	Trf	12,033	1,799
Inhibitor of carbonic anhydrase	Ica	8,269	2,028
Ferritin heavy chain	Fth1	9,508	1,038
Ceruloplasmin	Cp	24,744	4,352
Long chain fatty acid CoA ligase 3	Acsl3	12,898	0.947
Long chain fatty acid CoA ligase 4	Acsl4	4,554	0.683

Color code:
Ferroptosis
Fatty acid biosynthesis
Iron homeostasis

Subnetwork D

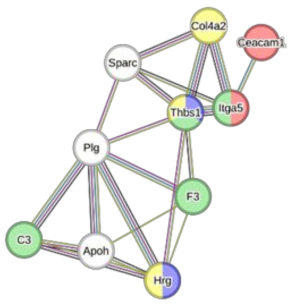

Protein name	Gene name	-Log pvalue	Ratio DNBS vs SHAM
Collagen alpha 2 IV chain	Col4a2	8,942	0.707
Carcinoembryonic antigen-related cell adhesion molecule 1	Ceacam1	3,692	0.653
Thrombospondin-1	Thbs1	11,008	5,016
Integrin alpha 5	Itga5	9,219	0.737
SPARC	Sparc	7,938	1,043
Plasminogen	Plg	15,263	2,315
Beta 2 glycoprotein 1	Apoh	10,913	1,326
Tissue factor	F3	6,476	1,324
Histidine rich glycoprotein	Hrg	15,330	4,413
Complement C3	C3	14,564	1,572

Color code:
Regulation of endothelial cell chemotaxis
Wound healing, spreding of cells
Positive regulation of angiogenesis
Angiogenesis

Table S8 - Proteins contributing to the predicted inhibition of the "Bleeding" function, as identified by Ingenuity Pathway Analysis (IPA) in the Diseases and Functions category. The table lists, for each protein, its expression log ratio, direction of regulation, and related biological findings. Proteins displayed in Figure 7 correspond to those included in this analysis.

© 2000-2025 QIAGEN. All rights reserved.

ID	Genes in dataset	Prediction (based on measurement direction)	Expr Log Ratio	Findings
S100a9	S100A9	Increased	5,929	Increases (0)
Fgb	FGB	Affected	5,093	Affects (1)
Thbs1	THBS1	Decreased	5,017	Decreases (2)
Fga	FGA	Decreased	4,877	Decreases (10)
Fgg	FGG	Decreased	4,711	Decreases (5)
Mpo	MPO	Increased	3,833	Increases (3)
Clu	CLU	Increased	3,49	Increases (1)
C4b	C4A/C4B	Affected	2,834	Affects (0)
Plg	PLG	Decreased	2,316	Decreases (181)
Fn1	FN1	Decreased	2,099	Decreases (2)
Car1	CA1	Affected	1,954	Affects (0)
F2	F2	Decreased	1,819	Decreases (77)
Trf	TF	Affected	1,799	Affects (1)
C3	C3	Affected	1,572	Affects (7)
Serpina1a	SERPINA1	Affected	1,458	Affects (0)
F3	F3	Decreased	1,325	Decreases (7)
F13a1	F13A1	Decreased	1,16	Decreases (6)
Alb	ALB	Decreased	0,847	Decreases (4)
Prdx6	PRDX6	Affected	0,832	Affects (1)
Egfr	EGFR	Affected	0,765	Affects (5)
Col18a1	COL18A1	Decreased	0,748	Decreases (1)
Apoe	APOE	Increased	0,726	Increases (3)
Col4a2	COL4A2	Affected	0,708	Affects (14)
Col4a1	COL4A1	Affected	0,679	Affects (6)
Ripk3	RIPK3	Decreased	0,649	Decreases (1)
Pkp2	PKP2	Decreased	0,638	Decreases (2)
Efemp2	EFEMP2	Decreased	0,616	Decreases (1)
Por	POR	Decreased	0,557	Decreases (1)
Ptgis	PTGIS	Increased	0,552	Increases (1)
Anxa1	ANXA1	Affected	0,549	Affects (1)
Psmb5	PSMB5	Affected	0,527	Affects (4)
Flna	FLNA	Increased	-0,503	Decreases (1)
Tuba1a	TUBA1A	Affected	-0,515	Affects (2)
Entpd1	ENTPD1	Decreased	-0,539	Increases (1)
Csk	CSK	Increased	-0,558	Decreases (1)
Adipoq	ADIPOQ	Increased	-0,587	Decreases (1)
Btk	BTK	Decreased	-0,965	Increases (1)
Muc2	MUC2	Increased	-0,992	Decreases (1)
Car3	CA3	Affected	-1,082	Affects (0)

Figure S5 - Graphical representation of the 'Inflammation of organ' pathway for which IPA predicts significant inhibition with a Z-SCORE value of -1.698. The set of genes up (red) and down (green) regulated to support the generated hypothesis are shown at the ends of the wheel graph.

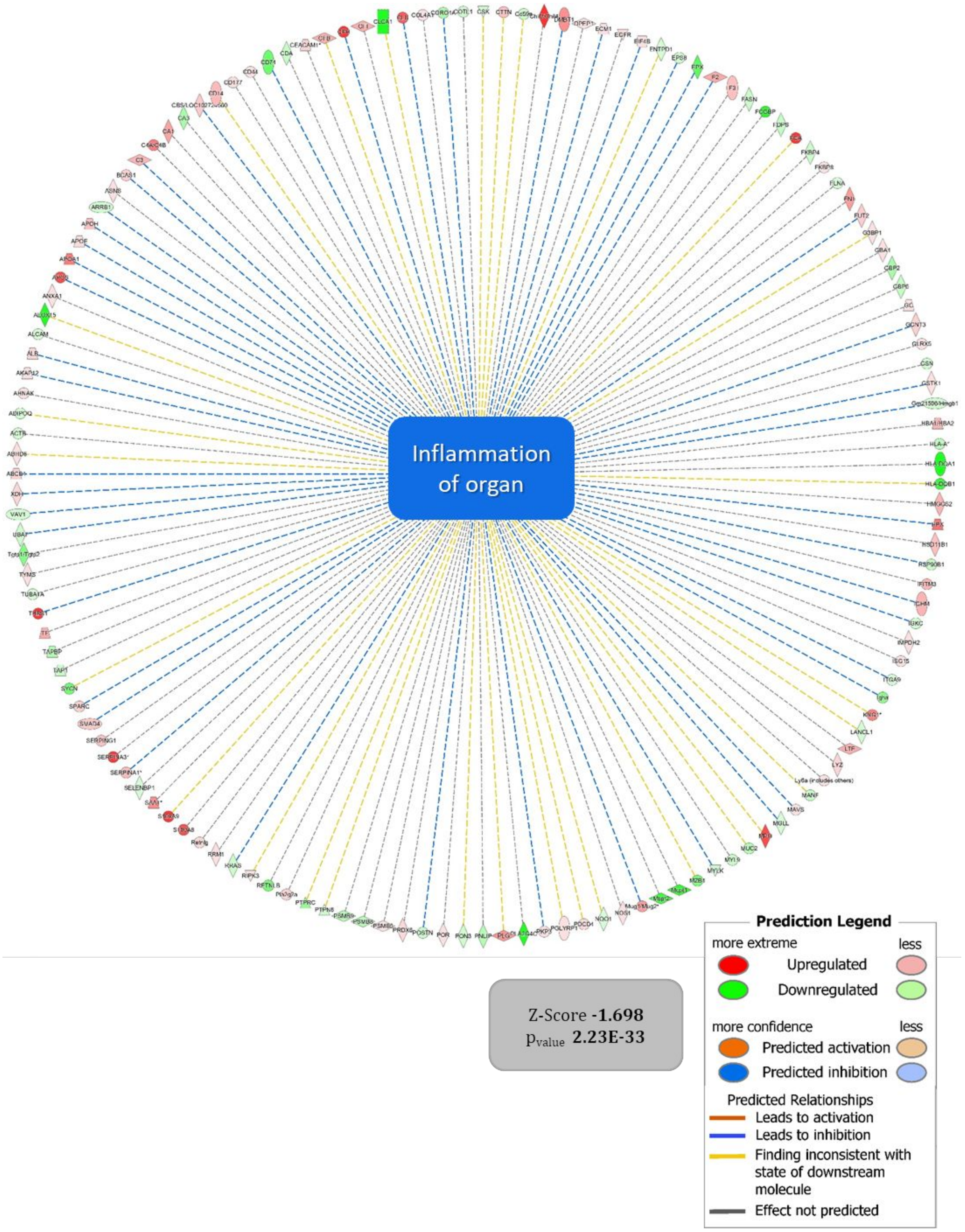

Table S9 - Proteins contributing to the predicted activation of the "Inflammation of organ" function, as identified by Ingenuity Pathway Analysis (IPA) in the Diseases and Functions category. The table lists, for each protein, its expression log ratio, direction of regulation, and related biological findings. Proteins displayed in Figure S5 correspond to those included in this analysis.

© 2000-2025 QIAGEN. All rights reserved.

ID	Genes in dataset	Prediction (based on measurement direction)	Expr Log Ratio	Findings
S100a9	S100A9	Increased	5,929	Increases (0)
Serpina3n	SERPINA3	Affected	5,568	Affects (0)
Thbs1	THBS1	Decreased	5,017	Decreases (7)
Fga	FGA	Increased	4,877	Increases (3)
Cfh	CFH	Decreased	4,774	Decreases (0)
Chil3	Chil3/Chil4	Affected	4,34	Affects (1)
Mpo	MPO	Increased	3,833	Increases (6)
S100a8	S100A8	Affected	3,818	Affects (13)
Apcs	APCS	Decreased	3,778	Decreases (11)
Clu	CLU	Decreased	3,49	Decreases (7)
Hpx	HPX	Decreased	2,989	Decreases (4)
C4b	C4A/C4B	Affected	2,834	Affects (0)
Apoa1	APOA1	Decreased	2,812	Decreases (3)
Kng1	KNG1	Increased	2,719	Increases (0)
Saa1	SAA1	Affected	2,427	Affects (0)
Dmbt1	DMBT1	Decreased	2,345	Decreases (1)
Plg	PLG	Increased	2,316	Increases (7)
Mug1	Mug1/Mug2	Decreased	2,148	Decreases (2)
Fn1	FN1	Affected	2,099	Affects (5)
Car1	CA1	Affected	1,954	Affects (2)
F2	F2	Decreased	1,819	Decreases (46)
Trf	TF	Affected	1,799	Affects (2)
Ltf	LTF	Affected	1,752	Affects (2)
Cfi	CFI	Affected	1,726	Affects (1)
Cfb	CFB	Increased	1,721	Increases (9)
Hba-a1	HBA1/HBA2	Affected	1,598	Affects (0)
Hmgcs2	HMGCS2	Affected	1,59	Affects (5)
Ifitm3	IFITM3	Affected	1,583	Affects (0)
Ighm	IGHM	Decreased	1,582	Decreases (13)
C3	C3	Decreased	1,572	Decreases (49)
Serpina1a	SERPINA1	Decreased	1,458	Decreases (0)
Cd14	CD14	Increased	1,438	Increases (4)
Cttn	CTTN	Increased	1,339	Increases (2)
Hsd11b1	HSD11B1	Affected	1,33	Affects (1)
Apoh	APOH	Decreased	1,326	Decreases (5)
F3	F3	Affected	1,325	Affects (1)
Serping1	SERPING1	Affected	1,32	Affects (1)
Smad4	SMAD4	Decreased	1,198	Decreases (6)
Cbs	CBS/LOC102724560	Decreased	1,116	Decreases (0)
Fut2	FUT2	Decreased	1,051	Decreases (0)
Sparc	SPARC	Decreased	1,043	Decreases (7)
Bcas1	BCAS1	Decreased	0,987	Decreases (1)
Gcnt3	GCNT3	Decreased	0,977	Decreases (2)
Abcb1a	ABCB1	Decreased	0,969	Decreases (0)
Pla2g2a	Pla2g2a	Affected	0,962	Affects (0)

Ceacam2	CEACAM1	Affected	0,933	Affects (0)
Lyz2	LYZ	Affected	0,912	Affects (0)
Retnlg	Retnlg	Affected	0,871	Affects (1)
Alb	ALB	Decreased	0,847	Decreases (9)
Xdh	XDH	Decreased	0,844	Decreases (7)
Prdx6	PRDX6	Affected	0,832	Affects (1)
Pdcd4	PDCD4	Increased	0,799	Increases (2)
Egfr	EGFR	Affected	0,765	Affects (32)
Apoe	APOE	Decreased	0,726	Decreases (11)
Cd177	CD177	Affected	0,702	Affects (0)
Isg15	ISG15	Affected	0,7	Affects (3)
Col4a1	COL4A1	Affected	0,679	Affects (111)
Rrm1	RRM1	Affected	0,674	Affects (6)
Cd44	CD44	Affected	0,672	Affects (19)
Ecm1	ECM1	Decreased	0,668	Decreases (5)
Glrx5	GLRX5	Affected	0,65	Affects (1)
Ripk3	RIPK3	Increased	0,649	Increases (5)
Gstk1	GSTK1	Decreased	0,645	Decreases (2)
Fkbp8	FKBP8	Affected	0,636	Affects (1)
Nos1	NOS1	Affected	0,631	Affects (1)
Asns	ASNS	Affected	0,601	Affects (1)
Akap12	AKAP12	Decreased	0,571	Decreases (1)
Pglyrp1	PGLYRP1	Increased	0,562	Increases (2)
Pkp3	PKP3	Decreased	0,559	Decreases (4)
Tyms	TYMS	Affected	0,557	Affects (58)
Por	POR	Affected	0,557	Affects (19)
Ahnak	AHNAK	Affected	0,551	Affects (2)
Anxa1	ANXA1	Affected	0,549	Affects (40)
Dpep1	DPEP1	Affected	0,544	Affects (16)
Eif4b	EIF4B	Decreased	0,539	Decreases (1)
Gc	GC	Affected	0,536	Affects (1)
Impdh2	IMPDH2	Affected	0,531	Affects (0)
Mavs	MAVS	Decreased	0,528	Decreases (4)
Psmb5	PSMB5	Affected	0,527	Affects (8)
Abhd6	ABHD6	Increased	0,526	Increases (4)
Gba	GBA1	Affected	0,525	Affects (3)
Ly6a	Ly6a (includes others)	Affected	0,511	Affects (1)
G3bp1	G3BP1	Increased	0,509	Increases (1)
Selenbp1	SELENBP1	Affected	-0,501	Affects (0)
Nqo1	NQO1	Increased	-0,502	Decreases (1)
Flna	FLNA	Affected	-0,503	Affects (2)
Alcam	ALCAM	Affected	-0,508	Affects (2)
Itga9	ITGA9	Decreased	-0,515	Increases (1)
Tuba1a	TUBA1A	Affected	-0,515	Affects (2)
Mgll	MGLL	Decreased	-0,527	Increases (2)
Entpd1	ENTPD1	Increased	-0,539	Decreases (4)
Lancl1	LANCL1	Increased	-0,551	Decreases (1)
Csk	CSK	Increased	-0,558	Decreases (2)
Vav1	VAV1	Decreased	-0,559	Increases (1)
Igkc	IGKC	Decreased	-0,562	Increases (2)
Actb	ACTB	Affected	-0,574	Affects (2)
Hmgb1	Gm21596/Hmgb1	Decreased	-0,581	Increases (0)
Arrb1	ARRB1	Decreased	-0,587	Increases (2)
Adipoq	ADIPOQ	Increased	-0,587	Decreases (17)
Eps8	EPS8	Decreased	-0,591	Increases (1)

Mylk	MYLK	Decreased	-0,595	Increases (1)
Uba7	UBA7	Decreased	-0,608	Increases (2)
Rras	RRAS	Decreased	-0,62	Increases (1)
Ptpn6	PTPN6	Increased	-0,62	Decreases (3)
Pon3	PON3	Increased	-0,626	Decreases (2)
Cotl1	COTL1	Affected	-0,64	Affects (2)
Fkbp4	FKBP4	Affected	-0,644	Affects (1)
Gsn	GSN	Affected	-0,647	Affects (1)
Fasn	FASN	Affected	-0,672	Affects (3)
Postn	POSTN	Decreased	-0,675	Increases (4)
Tap1	TAP1	Affected	-0,68	Affects (1)
Cd59a	Cd59a	Increased	-0,693	Decreases (7)
Manf	MANF	Increased	-0,705	Decreases (1)
Myl9	MYL9	Affected	-0,709	Affects (2)
Fdps	FDPS	Affected	-0,721	Affects (4)
Cda	CDA	Affected	-0,731	Affects (1)
Gbp4	GBP6	Affected	-0,737	Affects (0)
Hsp90b1	HSP90B1	Decreased	-0,748	Increases (3)
H2-K1	HLA-A	Affected	-0,867	Affects (8)
Psmb8	PSMB8	Affected	-0,911	Affects (4)
Pnlip	PNLIP	Affected	-0,932	Affects (1)
Psmb9	PSMB9	Affected	-0,943	Affects (1)
Tapbp	TAPBP	Affected	-0,978	Affects (1)
Muc2	MUC2	Increased	-0,992	Decreases (2)
Coro1a	CORO1A	Decreased	-1,056	Increases (5)
Car3	CA3	Affected	-1,082	Affects (2)
Gbp2	GBP2	Affected	-1,099	Affects (0)
Ptprc	PTPRC	Increased	-1,389	Decreases (14)
Igha	Igha	Decreased	-1,503	Increases (1)
Tgtp1	Tgtp1/Tgtp2	Affected	-1,52	Affects (2)
Mzb1	MZB1	Increased	-1,754	Decreases (1)
Retnlb	RETNLB	Affected	-1,778	Affects (0)
Sycn	SYCN	Increased	-1,834	Decreases (1)
Cd74	CD74	Decreased	-1,839	Increases (7)
Epx	EPX	Decreased	-2,068	Increases (1)
Mcpt2	Mcpt2	Affected	-2,485	Affects (1)
H2-Ab1	HLA-DQB1	Increased	-2,545	Decreases (0)
Mcpt1	Mcpt1	Affected	-2,632	Affects (1)
H2-Aa	HLA-DQA1	Affected	-2,815	Affects (0)
Fcgbp	FCGBP	Affected	-3,279	Affects (0)
Alox15	ALOX15	Increased	-3,367	Decreases (0)
Clca1	CLCA1	Increased	-4,176	Decreases (1)
Pla2g4c	PLA2G4C	Affected	-8,262	Affects (1)

Figure S6 - Graphical representation of the 'Immune response of neutrophils' pathway for which IPA predicts significant inhibition with a Z-SCORE value of -1.698. The set of genes up (red) and down (green) regulated to support the generated hypothesis are shown at the ends of the wheel graph**.**

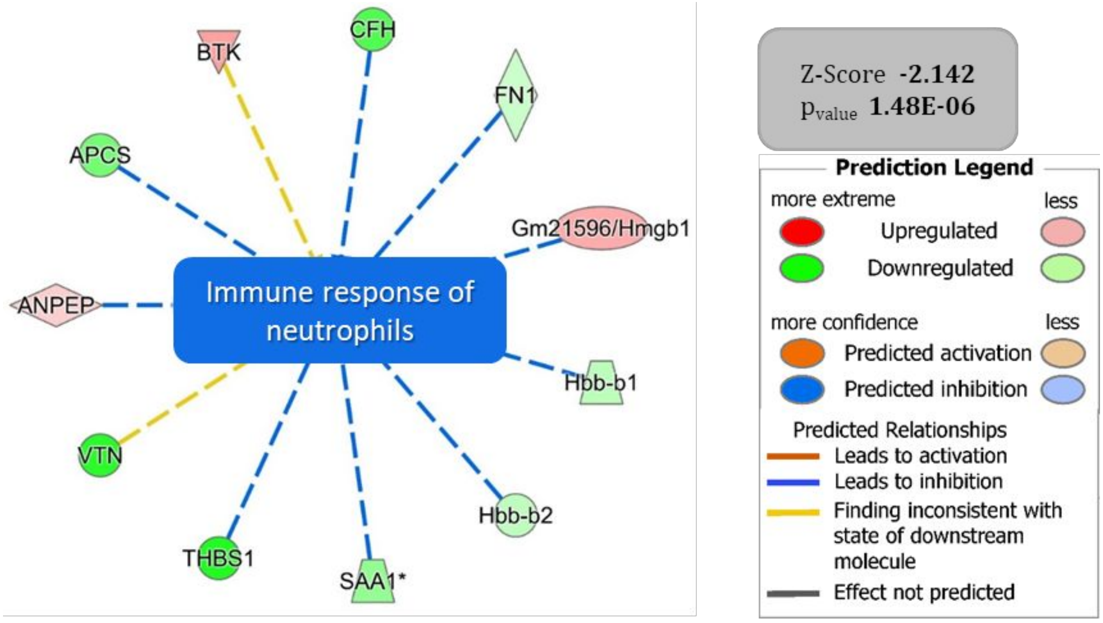

Table S10 - Proteins contributing to the predicted activation of the "Immune response of neutrophils" function, as identified by Ingenuity Pathway Analysis (IPA) in the Diseases and Functions category. The table lists, for each protein, its expression log ratio, direction of regulation, and related biological findings. Proteins displayed in Figure S6 correspond to those included in this analysis.

© 2000-2025 QIAGEN. All rights reserved.

ID	Genes in dataset	Prediction (based on measurement direction)	Expr Log Ratio	Findings
Btk	BTK	Increased	1,004	Increases (1)
Hmgb1	Gm21596/Hmgb1	Decreased	0,891	Decreases (0)
Anpep	ANPEP	Decreased	0,511	Decreases (1)
Fn1	FN1	Decreased	-0,605	Increases (2)
Hbb-b2	Hbb-b2	Decreased	-0,761	Increases (3)
Hbb-b1	Hbb-b1	Decreased	-0,802	Increases (3)
Saa1	SAA1	Decreased	-1,299	Increases (0)
Apcs	APCS	Decreased	-1,691	Increases (4)
Cfh	CFH	Decreased	-2,129	Increases (0)
Vtn	VTN	Increased	-2,608	Decreases (3)
Thbs1	THBS1	Decreased	-4,427	Increases (1)

Table S11 - Proteins contributing to the predicted activation of the Canonical Pathway identified as "Acute Phese Signaling" by Ingenuity Pathway Analysis (IPA). The table reports, for each protein, gene symbol, expression value (log ratio and p-value), subcellular localization, functional family and biomarker application(s). Proteins shown correspond to those supporting the activation of "Acute Phese Signaling" reported in Figure 9.

© 2000-2025 QIAGEN. All rights reserved.

Symbol	Entrez Gene Name	Gene Symbol - human (HUGO / HGNC / Entrez Gene)/Gene Symbol - mouse (Entrez Gene)	Expr p-value	Expr Log Ratio	Expected	Location	Type(s)	Biomarker Application(s)
A2M		A2m	6,58E-08	-1,287	Up	*Other*	*other*	
AMBP	alpha-1-microglobulinbikunin precursor	Ambp	0,00115	-0,709	Down	*Extracellular Space*	*transporter*	*safety,unspecified application*
APCS	amyloid P component, serum	Apcs	0,000403	-1,691	Up	*Extracellular Space*	*other*	*unspecified application*
CP	ceruloplasmin	Cp	0,00000203	-1,916	Up	*Extracellular Space*	*enzyme*	*efficacy*
FGA	fibrinogen alpha chain	Fga	6,87E-09	-2,849	Up	*Extracellular Space*	*other*	*diagnosis,unspecified application*
FGB	fibrinogen beta chain	Fgb	1,03E-08	-2,953	Up	*Extracellular Space*	*other*	*unspecified application*
FGG	fibrinogen gamma chain	Fgg	5,84E-09	-2,818	Up	*Extracellular Space*	*other*	
FN1	fibronectin 1	Fn1	0,032	-0,605	Up	*Extracellular Space*	*other*	*diagnosis,efficacy,prognosis,unspecified application*
FTL		Ftl1	0,0000342	0,654	Up	*Other*	*other*	
HNRNPK	heterogeneous nuclear ribonucleoprotein K	Hnrnpk	0,00000294	0,646	Up	*Nucleus*	*other*	*prognosis*
HP		Hp	0,00407	-2,559	Up	*Other*	*other*	
IL1RN	interleukin 1 receptor antagonist	Il1rn	0,00344	0,6	Up	*Extracellular Space*	*cytokine*	*efficacy*
ITIH2	inter-alpha-trypsin inhibitor heavy chain 2	Itih2	0,00264	-0,843		*Extracellular Space*	*other*	
ITIH3	inter-alpha-trypsin inhibitor heavy chain 3	Itih3	0,0102	-0,759		*Extracellular Space*	*other*	
ITIH4	inter-alpha-trypsin inhibitor heavy chain 4	Itih4	0,00185	-3,091		*Extracellular Space*	*other*	
PLG	plasminogen	Plg	0,000155	-1,004	Up	*Extracellular Space*	*peptidase*	*diagnosis,unspecified application*

SAA1		Saa1	0,000145	-1,299		*Other*	*other*	
SERPINA1		Serpina1d	0,00000471	-2,2	Up	*Other*	*other*	
SERPINA3		Serpina3n	5,81E-08	-3,534	Up	*Other*	*other*	
SERPINF2	serpin family F member 2	Serpinf2	0,00129	-0,827		*Extracellular Space*	*other*	
TTR	transthyretin	Ttr	0,000117	0,955	Down	*Extracellular Space*	*transporter*	*diagnosis,efficacy,prognosis,unspecified application*

Figure S7 - List of sub-networks of significantly up-regulated proteins (DNBS_TAP vs. DNBS comparison matrix), united by their involvement in specific biological processes; below the list of protein involved is the color code that associates each gene product with the GO (gene ontology annotation) used for functional enrichment.

Subnetwork E

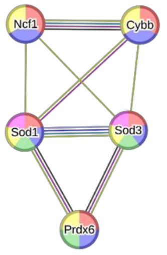

Protein name	*Gene name*	*-Log pvalue*	*Ratio DNBS_TAP vs DNBS*
Peroxiredoxin-6	Prdx6	6,886	1,394
Superoxide dismutase 3	*Sod3*	6,262	0.565
Superoxide dismutase 1	*Sod1*	4,952	0.510
Neutrophil cytosol factor 1	*Ncf1*	2,421	0.609
Cytochrome b-245 heavy chain	*Cybb*	3,088	0.575
Color code:			
Reactive oxygen species metabolic process			
Wound Cellular response to chemical stimulus			
Antioxidant activity			
Oxidoreductases activity			
Oxidative stress response			

Table S11

Comprehensive list of proteins significantly modulated in at least one of the three pairwise comparisons: **DNBS vs CTR**, **DNBS_TAP vs DNBS**, and **DNBS_TAP vs CTR**. Proteins were selected based on their statistical significance within each comparison and subsequently merged into a unified matrix using an *in-house* script specifically designed to ensure a consistent cross-condition comparison. It should be noted that not all proteins listed are significantly modulated across all three contrasts; rather, the resulting matrix represents the union of all proteins displaying significant modulation in at least one comparison.

This integrative approach was implemented to obtain a complete and harmonized dataset suitable for downstream analyses in *Ingenuity Pathway Analysis (IPA),* including *Biomarker Comparison* and multi-condition enrichment studies. For each protein, identified by its gene name, both the *p-value* and *log_2 ratio (fold-change)* are reported for the three comparisons.

Color coding has been applied to facilitate visual interpretation: **red shading** denotes proteins showing biologically relevant regulation ($|\log_2 \text{ratio}| > 0.5$), with **upregulated proteins in red** and **downregulated proteins in green**. Within the *p-value* columns, **non-significant values ($p > 0.05$)** are also highlighted in red to enable rapid distinction of statistically robust variations.
